# Human-Specific Organization of Proliferation and Stemness in Squamous Epithelia: A Comparative Study to Elucidate Differences in Stem Cell Organization

**DOI:** 10.3390/ijms26073144

**Published:** 2025-03-28

**Authors:** Ashlee Harris, Kaylee Burnham, Ram Pradhyumnan, Arthi Jaishankar, Lari Häkkinen, Rafael E. Góngora-Rosero, Yelena Piazza, Claudia D. Andl, Thomas Andl

**Affiliations:** 1 Burnett School of Biomedical Sciences, University of Central Florida, Orlando, FL 32826, USAburnhamkb@gmail.com (K.B.); ra370988@ucf.edu (R.P.); euraffa@gmail.com (R.E.G.-R.);; 2 Department of Oral Biological and Medical Sciences, Faculty of Dentistry, University of British Columbia, Vancouver, BC V6T 1Z1, Canada; lhakkine@dentistry.ubc.ca; 3 College of Medicine, University of Central Florida, Orlando, FL 32827, USA

**Keywords:** squamous epithelial biology, esophageal stem cells, spatial biology, multiplex immunofluorescence, comparative biology, MECP2, XPC

## Abstract

The mechanisms that influence human longevity are complex and operate on cellular, tissue, and organismal levels. To better understand the tissue-level mechanisms, we compared the organization of cell proliferation, differentiation, and cytoprotective protein expression in the squamous epithelium of the esophagus between mammals with varying lifespans. Humans are the only species with a quiescent basal stem cell layer that is distinctly physically separated from parabasal transit-amplifying cells. In addition to these stark differences in the organization of proliferation, human squamous epithelial stem cells express DNA repair-related markers, such as MECP2 and XPC, which are absent or low in mouse basal cells. Furthermore, we investigated whether the transition from basal to suprabasal is different between species. In humans, the parabasal cells seem to originate from cells detaching from the basement membrane, and these can already begin to proliferate while delaminating. In most other species, delaminating cells have been rare or their proliferation rate is different from that of their human counterparts, indicating an alternative mode of how stem cells maintain the tissue. In humans, the combination of an elevated cytoprotective signature and novel tissue organization may enhance resistance to aging and prevent cancer. Our results point to enhanced cellular cytoprotection and a tissue architecture which separates stemness and proliferation. These are both potential factors contributing to the increased fitness of human squamous epithelia to support longevity by suppressing tumorigenesis. However, the organization of canine oral mucosa shows some similarities to that of human tissue and may provide a useful model to understand the relationship between tissue architecture, gene expression regulation, tumor suppression, and longevity.

## 1. Introduction

Within the class of mammalia, few species exhibit a maximum lifespan above 100 years [1]. Humans belong to this small group of mammals with extreme longevity. The molecular basis of longevity in mammals remains poorly understood, but it is widely believed that all organs and tissues in long-lived organisms must be optimized, as even the weakest link can determine overall morbidity [2].

A contributor to morbidity is cancer, which primarily occurs in self-renewing epithelia and is associated with stem cell proliferation rates [3]. Aging in such epithelia leads to loss of repair capacity, cellular senescence, and clonal selection of cell populations that may become malignant [4]. To maintain these epithelia and prevent them from becoming the “weakest link,” the epithelial stem cells likely have enhanced protection and increased resilience programs [5].

Different organs or tissues may employ distinct mechanisms to promote longevity, and each mammalian order—or even each mammalian species—may have evolved unique strategies to address the challenges of long life. For example, the liver cells of long-lived mammals display a coordinated reprogramming of gene expression at a genome-wide scale, including reduced expression of genes involved in the metabolism of saturated and unsaturated fatty acids [6]. In contrast, bats, which are exceptionally long-living considering their small size, exhibit adaptations at the proteome level. Comparative analyses of bat and mouse liver cells suggest that bats have evolved enhanced protein stability, resistance to protein oxidation, and overall improved protein homeostasis [7].

Longevity in mammals is linked to improved DNA repair, protein quality control, and immune function [8,9,10,11,12]. However, beyond enhancing repair and quality control, a critical protective mechanism involves avoiding processes that elevate the risk of mutations and cancer. Because DNA replication and mitosis are among the riskiest processes for introducing mutations [3,13], fibroblasts from some small long-lived rodents exhibit markedly reduced growth rates compared to those from short-lived species [14]. That study further indicated that strategies to achieve longevity vary significantly: large rodents tend to rely on replicative senescence, whereas small rodents modulate cell division rates as an adaptive mechanism [14].

Organizing the germinative compartment of the epithelium into stem cells and transit-amplifying cells (also known as originally as transient amplifying cells [15] or transitory proliferative cells [16]) can maximize both cell renewal and the protection of genetic integrity [5,17]. This physical separation is seen in human squamous epithelia, such as those found in the oral cavity, esophagus, anus, vagina, and ectocervix, and is unique among studied mammalian species.

To understand the potential role of this distinct organization in longevity, cancer-protection, and its fate in cancer development, we compared the expression of genes associated with cellular protection against DNA damage in human squamous epithelial cells to that in other mammalian species. Using high-plex immunofluorescence staining that we validated for cross-species analysis and image segmentation in conjunction with single-cell RNA sequencing data, we analyzed the differences in squamous epithelial stem cell biology between humans and 15 other mammals (Supplementary Table S1). Our specific focus was to detect differences between short-lived and long-lived species, with emphasis on cellular protection mechanisms. The toolset included antibodies and multiplexing that can characterize tissue architecture and cell types and adaptive changes in morphology and tissue architecture that may drive longevity (references and basic information on these proteins are provided in Supplementary Table S1). This approach is based on the observations outlined by Carroll [18] that alterations in gene expression, rather than DNA and protein sequence changes, drive adaptations including longevity [6]. Thus, our approach interrogated both changes in protein expression levels and patterns as well as tissue architecture.

Such a detailed, two-pronged approach has led us to identify a quiescent basal cell layer in humans and define its gene expression pattern using small tissue samples. Two markers, XPC and MECP2, stood out. XPC is an essential component of the DNA damage control machinery and its loss in humans leads to dramatically reduced life expectancy [19,20,21]. Its role in squamous epithelial outside of the skin has not been explored in detail. A protein that can interact with XPC is MECP2 [19]. It has also become associated with the protection of genomic integrity and its mutation results in reduced life expectancy in mice and men [22,23]. We identified both as markers of quiescent stem cells of human squamous epithelia and show that they interact specifically in these cells. Our results indicate that human basal cells exhibit elevated expression of cytoprotective proteins, including XPC and MECP2, which is not observed to the same extent in the basal cells of other species. Moreover, this elevated expression is lost in precancerous and cancerous lesions, supporting the hypothesis that MECP2/XPC expression and basal cell quiescence may play a tumor-suppressive role. However, dog squamous epithelia have some features of proliferation organization that could make them useful models of the human condition and for oral cancer studies.

## 2. Results

### 2.1. Establish a Set of Antibodies Suitable for Cross-Species Analysis of Proliferation and “Stemness”

To establish a multispecies multiplex immunofluorescence comparison, we evaluated a set of antibodies that define proliferation, differentiation, stemness, and cell outline in squamous epithelia. We initially tested 29 antibodies (Supplementary Table S1). In brief, proliferation marker antibodies such as PCNA, phosphor-serine 10 Histone H3 (pH3), and MCM2 worked nearly universally in the mammals we tested. To define epithelial cells and their outlines or cell bodies, we examined antibodies against CDH1 (E-cadherin), KRT14, CTNNB1 (beta-catenin), and CD44. With few exceptions, all these antibodies work well in mammals, but only the anti-CDH1 antibody also detected CDH1 in chicken.

### 2.2. Basal-Suprabasal Cell Dichotomy in Humans and Absent in Other Analyzed Species: Basal Cell Quiescence and Accompanying MECP2 and XPC Expression Are Human Features

**Figure 1:**
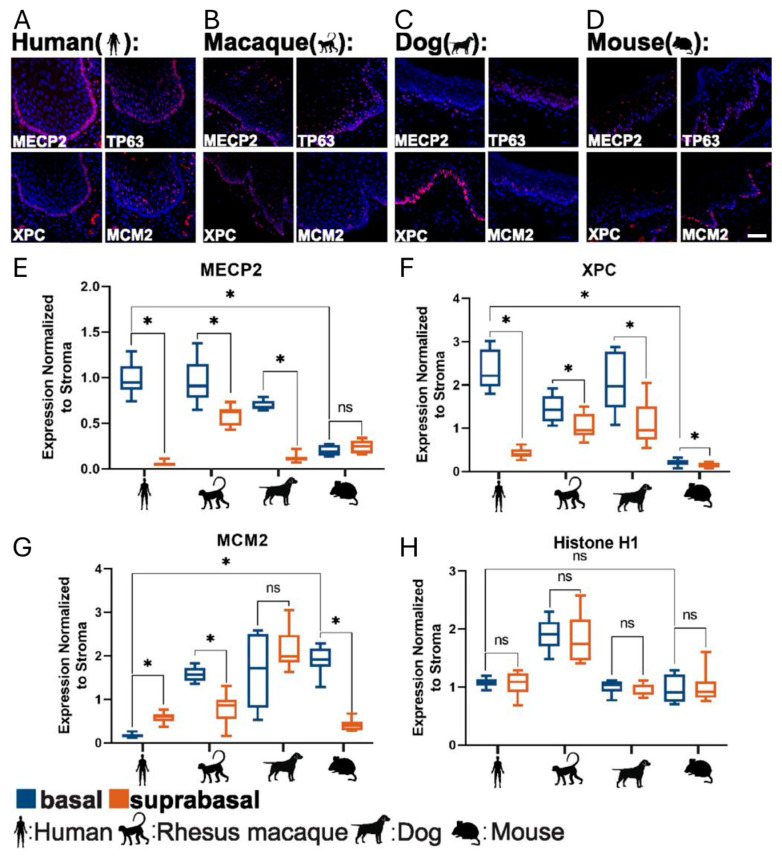
Low proliferation and high MECP2 and XPC expression are most pronounced in humans and absent in mice. (**A**–**D**). Fluorescent images of oral squamous epithelial from four different species showing MECP2, TP63, XPC (basal cell markers), and MCM2 (proliferation) expression. (**E**–**H**). The comparison between basal and suprabasal cells shows that in mice there is no difference in MECP2, XPC and Histone H1 expression, but mouse suprabasal cells are non-proliferative. In humans and dogs, MCM2 expression is mainly found in suprabasal cells. Data shown are from manual multiplex stainings and manual cell segmentation in Leica LASX. Data were exported to Microsoft Excel Version 16.95.1 and Graphpad. * indicates significant difference of *p* < 0.5 while ns indicates no statistical difference. White bar in immunofluorescence image represents 50 µm.

With a set of broadly applicable antibodies on hand, we determined quantitative and qualitative differences in protein expression. MECP2 and XPC are novel markers of the basal cell layer, especially in humans, and their specific basal expression in certain mammals prompted us to further analyze them. They are also unique in the sense that related proteins do not exhibit basal specific expression (Supplementary Figure S1) and XPC binding partners are expressed basally while other DNA damage related proteins are suprabasally expressed (Supplementary Figure S2). Therefore, we hypothesized that their analysis may offer new insights into differences in epithelial organization in mammals and how DNA damage repair is specifically managed in quiescent basal stem cells. To put their expression into context, we also assessed expression of TP63 (general epithelial marker with preferences to basal cell expression), Histone H1 (expressed in all cells and therefore useful for normalization), and MCM2 (a reliable proliferation marker). References for protein functions and protein classification are found in Supplementary Table S1. Both MECP2 and XPC are barely expressed in mouse squamous epithelia (ca. 5 and 11-fold lower in mouse compared to human basal cells, respectively, Figure 1 and Supplementary Table S2). As expected, proliferation was low in this MECP2^high^ human basal cell layer (the quiescent stem cell layer), while it was high in MECP2^−^ parabasal cells (Figure 1A). On the other hand, most other mammalian species completely lacked a parabasal proliferative population and proliferation and stemness were restricted to the basal cell layer. The classic example for this organization can be found in rodents, where expression of a proliferation marker such as MCM2 is restricted to the basal cells (Figure 1D versus 1A, Figure 1H). Using a multiplex immunofluorescence staining technique (Supplementary Table S3) and measuring the fluorescent signal in each epithelial cell after manual cell segmentation, we analyzed MECP2, XPC, P63 (markers associated with the basal cell layer), MCM2 (proliferation marker) and histone H1 (normalizer) in esophageal and oral squamous epithelial cells (Figure 1). The differences in human epithelial organization are highlighted when comparing basal and suprabasal expression by measuring fluorescent intensities in individual cells and normalizing expression either to stromal cells (Figure 1E–H), using histone H1 (a marker expressed at similar levels in basal and suprabasal cells), or using unnormalized data (Supplementary Table S2). In humans, there is a pronounced and abrupt drop in expression of MECP2 and XPC from basal to suprabasal cells (Supplementary Table S2, ca. 16- and 5-fold, respectively) and humans express about 5-fold more MECP2 and 11-fold more XPC in their basal cells than mice do (Supplementary Table S2). This drop from basal to suprabasal cell expression is still present although attenuated in monkey (Figure 1B,E,F), and absent in rodents such as rats and mice (Figure 1C–F). Interestingly, out of the species tested, dogs showed a surprising resemblance to humans, suggesting an even higher similarity between dog and human tissue organization than human and monkey. To make our analysis more robust and to control for differences between species and tissue quality, we used histone H1 to serve as a reference value to normalize gene expression (similar to approaches used in qRT-PCR and Western blotting). All the 22 analyzed markers except for TP63 were also expressed in stromal cells. Therefore, we also used these stromal cells as internal normalizers for the epithelial expression of MECP2, XPC, MCM2, and Histone H1. Thereby, we performed normalization based on a reference protein (histone H1) and based on reference cells (stromal cells). The noted expression of the proteins in the stromal cells also suggests that low or absent expression of a marker in the epithelium in the samples is unlikely to be an artifact.

In addition to MECP2 and XPC, we also investigated the keratinocyte marker TP63 which shows generally the highest expression in basal cells (Supplementary Table S2). Normalization to histone H1 overall preserved this trend. Therefore, TP63 can be regarded as a squamous epithelial basal stem cell marker as it has been suggested in the literature [24].

In contrast to the widely used animal models, mice and rats, which only proliferate in the basal cell layer, we also included less conventional animal models such as pigs and dogs in our study to explore alternative models of squamous epithelial cell biology. Interestingly, the dog sample showed areas of basal cell quiescence reminiscent of a human epithelium suggesting that dogs may be suitable to explore characteristics of this cell layer in a way that is also relevant for humans. This observation is in line with our previous findings [25] and findings by Sa et al. [26] who also observed similarities regarding Ki-67 expression between humans and dogs.

### 2.3. Single-Cell Analysis of Basal Cells In Situ: Heterogeneity Within the Basal Cell Layer

**Figure 2:**
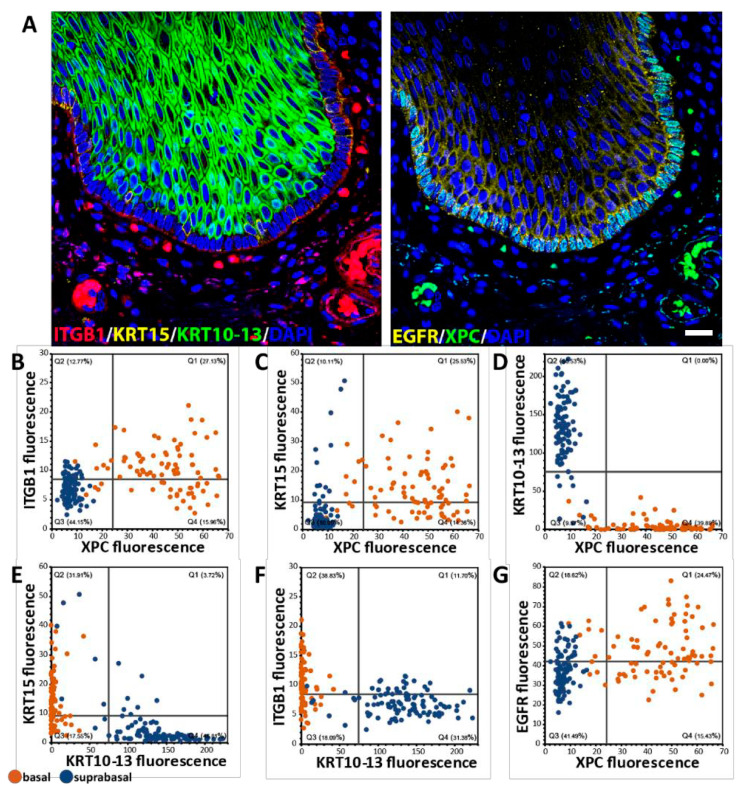
Human basal cell heterogeneity based on characteristic markers. (**A**)**.** Multiplex immunofluorescence staining of human esophageal epithelium exhibiting a distinctly basal cell expression of XPC. (**B**). A high percentage of basal cells (orange dots) are XPC and ITGB1 positive. (**C**). Although there is significant overlap between XPC and KRT15 expression in basal cells, subpopulations appear to be XPC, KRT15, or ITGB1 positive. (**D**). No XPC^+^ basal cell expresses differentiation marker KRT10 or 13. (**E**,**F**). Neither KRT15 nor ITGB1 are enriching basal cell populations as well as XPC; and both can show co-expression with KRT10/13. (**G**). EGFR is not a specific basal cell marker. Data are derived from manual multiplex stainings and manual cell segmentation (see Supplementary Table S3 for staining outline). Data were exported from Leica LASX and analyzed in Excel and Graphpad. White bar in immunofluorescence image represents 50 µm.

To expand on our previous description of squamous epithelia in multiple mammalian species by using highly multiplexed immunofluorescence stainings and single-cell analysis, we first established that single-cell analysis allows us to define the basal cell population in situ. In contrast to other approaches such as single-cell RNAseq or flow cytometry, such an in situ multiplex approach retains spatial information as well as cellular and tissue context [27,28]. Comparing the expression of traditional basal cell markers such as ITGB1, KRT15, and EGFR, we evaluated how well the novel basal cell marker XPC depicts basal cells, as indicated in Figure 2A. Using an antibody against KRT10/13 (absent in basal cells), ITGB1 and KRT15 (established basal cell markers) and EGFR (elevated in basal cells), we were able to define the XPC population at the individual cell level (Figure 2A). Identification of individual cells and plotting the expression values of the markers for each cell show that XPC^+^ cells are ITGB1^+^ (Figure 2B), KRT15^+^ (Figure 2C) and KRT10/13^neg.^ (Figure 2D). Using a manual definition of basal cells based on location and marker expression (highlighted in orange in Figure 2B–G), XPC^+^ cells were better able to define “basalness” than KRT15, ITGB1, or EGFR. Of the 85 manually defined basal cells in Figure 2, XPC^+^ identified 88.24%, ITGB1^+^ 67.06%, KRT15^+^ 63.53%, and EGFR^high^ 62.35% of basal cells; 100% of the cells were double-positive for XPC, and the other basal cell markers were basal cells. In summary, the multiplex approach allows us to better define cell populations in situ. Using XPC as an example of a previously unknown basal cell marker, we show that substantial heterogeneity within the basal cell population, regarding the expression of XPC and other basal markers, exists. For example, whether XPC^low^ basal cells represent a special basal subpopulation that is proliferative or is leaving the basal cell population is unclear.

### 2.4. Behavioral Heterogeneity in the Basal Cell Layer: Relationship Between Delamination and Proliferation

**Figure 3:**
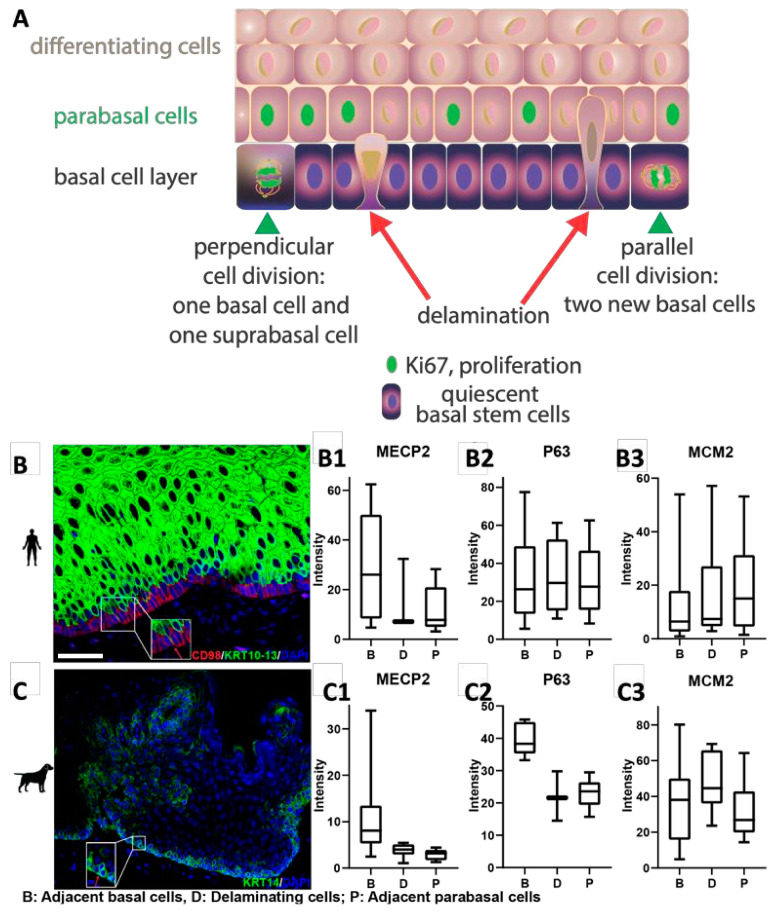
Quantification and characterization of delaminating cell populations in comparison to adjacent cells. (**A**). Schematic diagram of mechanisms to deliver cells to the suprabasal cell compartment. (**B,C**). Example of delaminating cells in human and dog epithelia, respectively. Inserts show exemplary delaminating cells at higher magnification. (**B1/C1**). Using the basal cell marker MECP2, delaminating cells (D) exhibit a pronounced drop in MECP2 expression compared to adjacent basal cells (B) reaching levels similar to adjacent parabasal cells (P). This observation is true for both humans and dogs. (**B2/C2**). In contrast to MECP2, p63 expression is not different in B, D and P cells in humans. However, in dogs, p63 expression drops like MECP2. (**B3/C3**). The intensity of MCM2 expression per B, D, and P cell does not change. White bar in (**A**) represents 50µm.

Although proliferation is low in the human basal cell layer, the parabasal and suprabasal cells must be replenished to maintain epithelial homeostasis. How this replenishment occurs is unclear (Figure 3A). Traditionally, supply of new cells to the suprabasal cell compartment has been discussed in terms of oriented cell divisions using terms such as planar, perpendicular, and asymmetric cell divisions with a focus on whether a basal cell produces a new basal cell or a suprabasal cell [29,30]. In contrast, delamination has been speculated to be of greater importance in adult squamous epithelia, such as mouse ear epidermis. However, in these experiments skin was exposed to a powerful depilatory cream that may have altered cellular behavior [31]. To obtain an overview of the basal cell replenishment in human squamous epithelia versus other species, we counted the number of delaminating cells and whether they were proliferating (Ki67 or MCM2 or PCNA positive). We defined delamination histologically (Figure 3B,C). In humans, only a few delaminating cells were proliferating based on Ki67 staining (Table 1); however, the numbers of MCM2^+^ and PCNA^+^ cells, both proliferation markers, indicates that 40% of the delaminating cells are potentially proliferative. On the other hand, there is a steep drop in the basal cell marker MECP2 in delaminating cells to levels seen in parabasal cells (Figure 3(B1)). In dogs, we observed a similar MECP2 expression drop in delaminating cells (Figure 3(C1)). However, in dogs -in contrast to humans- a similar phenomenon to the MECP2 expression drop can be observed for p63, whereas in humans p63 expression is maintained in delaminating cells (Figure 3(B2,C2)). Although most species showed a higher proliferation rate of delaminating cells than humans, a clear overall abundance of these cells was still found mainly in humans, dogs, and mice (Table 1; Supplementary Table S4). For dogs and mice and to some extent for humans, the findings resemble those in human epidermis by Wang et al. [32] who observed “transitional” basal cells that appear to be in the process of delaminating from the basal layer while proliferating. This suggests that in squamous epithelia, delaminating cells can start to proliferate while being in transition. However, in humans, most delaminating cells did not seem to be actively proliferating based on Ki67 staining, a marker that more narrowly identifies proliferating cells in comparison to PCNA. In summary, delamination is a common mechanism to supply new cells to the suprabasal cell compartment in many mammalian epithelia and may contribute to the basal cell heterogeneity we have observed (Figure 2). However, in humans, delamination may be more common than in other species and delamination is less frequently associated with proliferation.

### 2.5. Organization of the Basal Cell Layer in Different Species: Canine Oral Basal Cells Exhibit Two Different Types of Basal Cells

**Figure 4:**
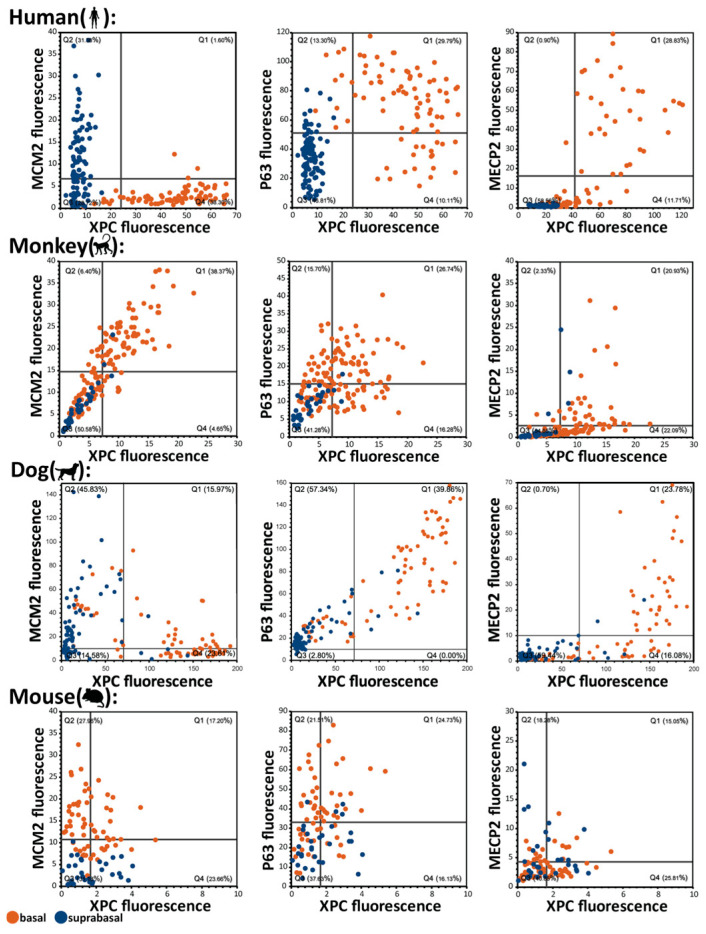
Characterization of basal cells versus suprabasal cells of multiple species using basal cell markers XPC, MECP2, p63 and proliferation marker MCM2. Manual multiplex stainings were analyzed by manually identifying cells and measuring their expression levels using Leica LASX version 3.7 software.

**Figure 5:**
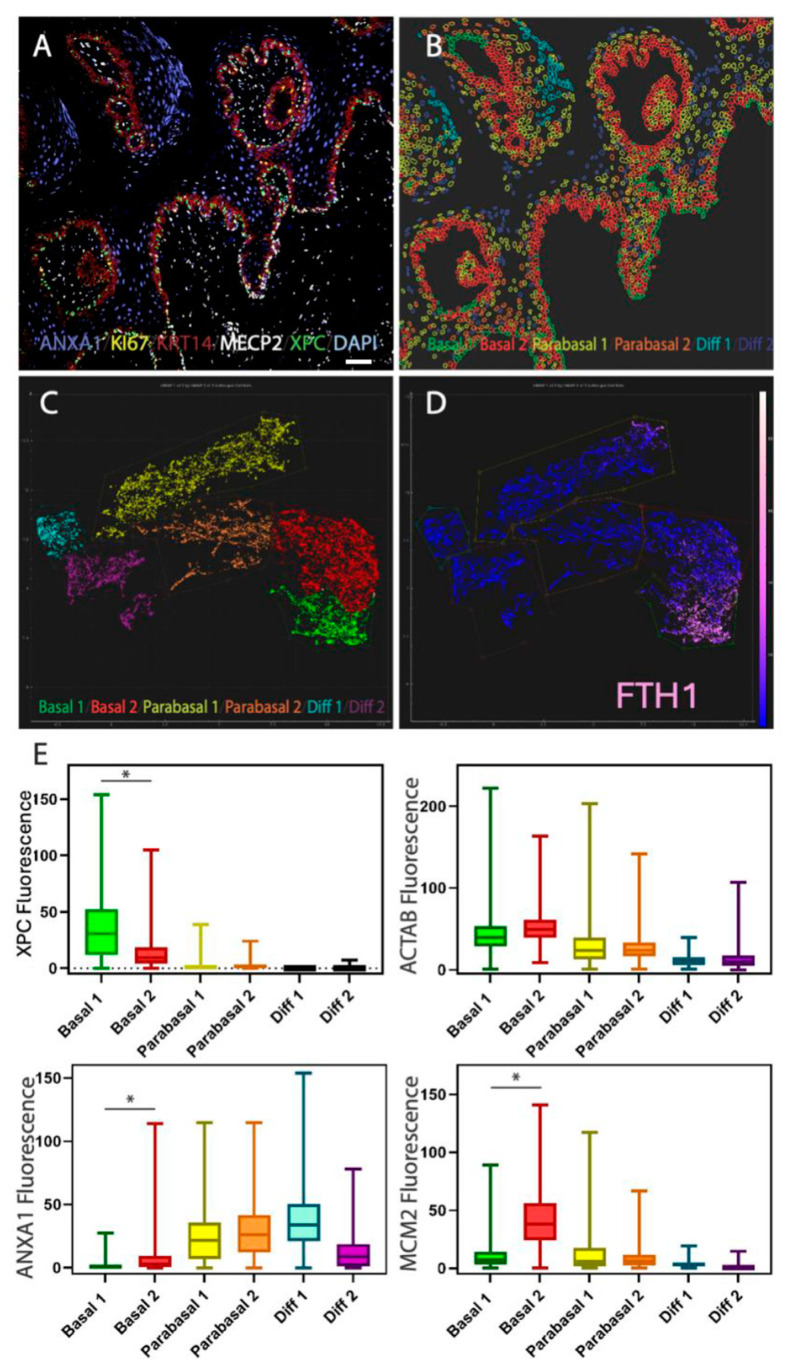
Analysis of canine basal cell populations and their similarities to the human condition. (**A**). Example of dog oral mucosa 16-plex multiplex stain showing 5 markers. (**B,C**). UMAP clustering of epithelial cells shows 6 cell populations including 2 basal cell populations (green and red). (**D**). Heatmap of FTH1 expression within UMAP shows enhanced expression in “green” basal cell population. (**E**). Definition of “green” basal cells (Basal 1) as cells similar to human basal cells. Data are derived from manual multiplex stainings (see Supplementary Table S5). Confocal images were exported and used in MACSiQView to cluster cell populations using UMAP. To generate box plots, data were exported from MACSiQView and analyzed in Graphpad. * indicates significant difference of *p* < 0.5 while ns indicates no statistical difference. White bar in (**A**) indicates 50 µm.

To clarify the differences between species in their basal cell characteristics, we used multiplex stainings and defined the relationship between individual markers focusing on human, monkey, dog, and mouse. In humans, there is a clear absence of proliferation in basal cells (represented by orange dots in the scatter plots in Figure 4 MCM2), however this separation of basalness and proliferation is reversed in mice. In monkeys, the basal cells show proliferation (based on marker MCM2) and expression of XPC can be associated with proliferation. In humans, expression of XPC is almost completely excluded from MCM2 positive cells. Therefore, mice and humans represent extremes of squamous epithelial organization. However, in the dog, as alluded to in Figure 1 and Figure 3, the features of basal cells are more complex than in the other analyzed species. To untangle this complexity, we performed a 16-plex manual multiplex stain (Supplementary Table S5 for antibodies and procedure) on normal dog oral mucosa (Figure 5A). Using the Miltenyi MACSiqView 1.3 software, we were able to group epithelial cells into 6 clusters using the UMAP tool (Figure 5B,C). Basal cells formed two clusters. One cluster (green cluster in Figure 5C) is characterized by high expression in the human quiescent basal cell marker FTH1, which is related to ferroptosis prevention. The anti-FTH1 antibody works well in both human and dog tissues. The other cluster is largely FTH1 negative (red cluster in Figure 5C,D). A closer inspection of both clusters and basal cell populations in dogs suggests that this FTH1-high “Basal 1” population corresponds to cells that resemble human quiescent basal cell layer stem cells (XPC^+^/ANXA1^−^/MCM2^low^) as shown in Figure 5E.

### 2.6. Comparison of scRNAseq Datasets Confirms Human and Mouse Differences in Basal Cell Proliferation and Proliferation-Associated TGFbeta, WNT, and SHH Pathways

**Figure 6:**
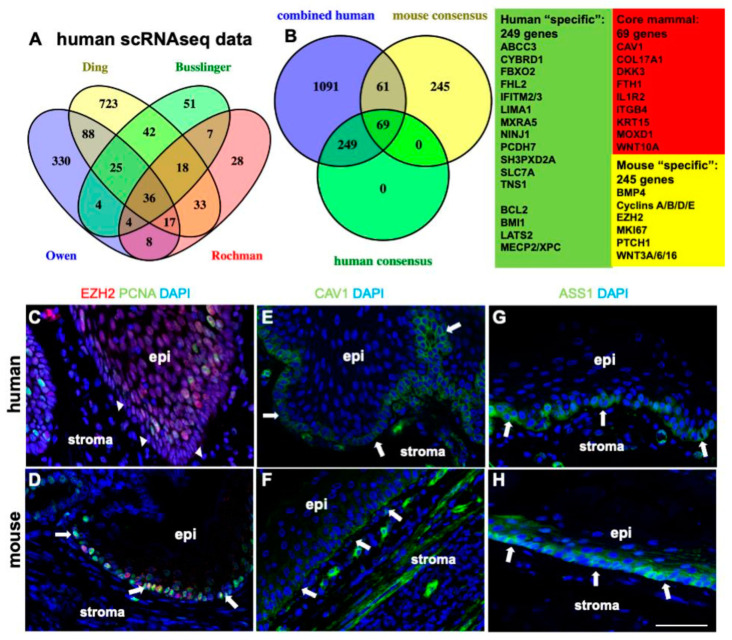
Determining human specific basal cell gene expression. (**A**). Venn diagram to identify human basal cell markers based on scRNAseq datasets. (**B**). Commonalities and differences in mouse and human basal cell markers: highlighting a role of NRF2 signaling, ferroptosis, and a lack of DNA repair related basal markers XPC and MECP2 in scRNAseq data. (**C**). In humans, basal cells (white arrowheads) express little EZH2. EZH2 (red) is mainly expressed in proliferating (PCNA^+^ green) human parabasal cells. (**D**). In contrast, in mice EZH2 (red) is expressed in proliferating (PCNA, green) basal cells (white arrows). (**E**,**F**). CAV1 is expressed in basal cells (white arrows) in both humans and mice. (**G**,**H**). ASS1 is expressed in basal cells (white arrows) in both humans and mice. White bar in (**H**) represents 50 µm and can be applied to (**C**–**F**) as well.

To define whether proliferation is the main difference between mouse and human basal cell layer cells, we next compared existing single-cell RNAseq (scRNA-seq) data from mouse and human squamous epithelia. Although the studies included in our comparison vary greatly in their basal cell marker sets and show limited overlap (Figure 6A; see Supplementary Table S6), key characteristics that consistently distinguish human from mouse basal cells are enhanced “TGF-beta regulation of extracellular matrix” “Cytokine Signaling” and “NRF2 Pathway”/”Ferroptosis” signatures. For examples of ferroptosis-related protein expression in the human basal cell layer, see Supplementary Figure S3 and Supplementary Table S7. As expected, the main characteristic of the 245 mouse-specific basal cell layer markers (Figure 6B) that are absent in humans, is their involvement in regulation of cell proliferation (e.g., KEGG term “Cell Cycle”; Supplementary Table S6). Beyond proliferation, the mouse data indicate enhanced Hedgehog and WNT Signaling Pathway signatures, as well as a small “Cysteine and methionine metabolism” related gene signature (Figure 6B, mouse “specific” genes include cyclins and other proliferation markers; Supplementary Table S6).

In addition, considering target genes of transcription factors from published ChIP datasets (ChEA database) human basal cells tend to have an overrepresentation of the anti-proliferative SMAD2/3/4 target genes [25] while mouse basal cells show an overrepresentation of proliferation associated FOXM1 target genes (Supplementary Table S6). Overall, these data confirm our previous findings ([5,25], Figure 1 and Figure 4) and corroborate a different organization of the mouse basal cell layer compared to the human basal cell layer.

### 2.7. XPC and MECP2 Interaction Is Restricted to Quiescent Basal Cells

A human enhanced “pathway” that does remain undetected in the scRNAseq comparison, likely due to either low mRNA expression or posttranscriptional regulation, is genome protection via elevated DNA repair. Two proteins, XPC and MECP2, stand out because of their role in DNA repair [20,33,34,35,36,37], their basal cell co-expression (see Figure 1 and Figure 4), and their direct interaction with each other [19]. Given that the pattern and intensity of MECP2/XPC co-expression differ between mice and humans (Figure 1, Figure 2, Figure 3 and Figure 4), we questioned whether this co-expression is a characteristic feature of human stem cells, contributing to their health and potentially playing a tumor-suppressive role by preventing squamous cell carcinoma and its precursors. We speculate that MECP2 and XPC expression is lost during tumorigenesis because of their putative keratinocyte stem cell–protective functions [38]. To test this hypothesis, we set out to determine: 1. Whether the expression and interaction of MECP2 and XPC are altered in precancerous lesions, and 2. at what stage the quiescent basal cell layer—putatively tumor-suppressive—is disrupted during the normal-precancer-cancer progression, and how this disruption affects basal marker expression.

**Figure 7:**
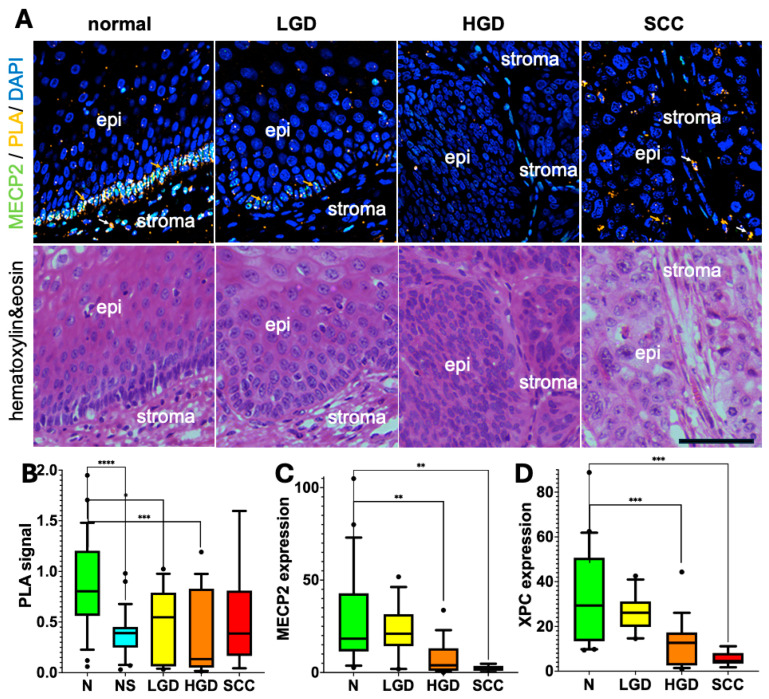
Proximity ligation assay (PLA)-based analysis of MECP2–XPC interactions. (**A**). Representative PLA examples depicting interactions specifically in human basal cells but loss in human esophageal dysplastic lesions and SCC. PLA signal occurs as orange dots and some of them are highlighted with orange arrows on epithelial cells. White arrows indicate stromal artifacts. To define basal cells several markers were stained subsequent to the PLA procedure including SLC3A2, KRT10/13, and Ki67 (see Supplementary Figure S4, which also contains information about control PLA experiments). Below the PLA image are hematoxylin and eosin (H&E) stains from the same area. Black bar in the H&E stain represents 50 µm. (**B**). Quantification of PLAs showing a significant reduction in PLA signal from normal basal cells (N) to suprabasal cells (NS) in normal squamous epithelial. A similar or even more pronounced drop in PLA signal is observed during the progression from normal (N) to squamous cell carcinoma (SCC). Student’s t test shows a statistical reduction in PLA signal between NB and the following states: NS, LGD (low grade dysplasia), HGD (high grade dysplasia), but not SCC. (**C**,**D**). MECP2 and XPC expression, respectively, are reduced similar to the PLA signal from normal to dysplasia and SCC. Student’s t test shows a statistical reduction in MECP2 as well as XPC expression between NB and the following states: HGD and SCC. Statistical significance: * *p* < 0.05, ** *p* < 0.01, *** *p* < 0.001, **** *p* < 0.0001. N, normal basal cells from 25 samples; NS, normal suprabasal cells from 25 samples; LGD, low-grade dysplasia basal cells from 14 lesions; HGD, basal like cells from 19 high-grade dyspalsias; SCC, basal like cells from 9 SCC samples. Analysis solely based on cells rather than averages for each sample are provided in Supplementary Figure S4. All samples from TMA BBS02011, imaged on a Leica SP5 microscope, analyzed in MACSiqView, and graphs generated in Prism.

If MECP2/XPC expression and basal cell quiescence are indeed tumor-suppressive (and linked to longevity), then we would expect a coordinated loss of MECP2, XPC, and basal cell quiescence during the transition to precancer. Since a key factor for stem cell quiescence is the reduction in replication errors, any impairment in this process could contribute to tumorigenesis.To explore these ideas, we performed a combination of proximity ligation assays (PLA) and MECP2/XPC immunofluorescence stainings to show interaction between MECP2 and XPC, specifically in normal human quiescent basal cells which become progressively lost during progression from normal to cancer (Figure 7A, Supplementary Table S8 for overview of procedure). In normal human squamous epithelia (in this case of the esophagus), PLA signal is restricted to basal cells (Figure 7A, quantification 7B). However, while XPC and MECP2 have strong signals in the basal cell layer, based on the PLA signal, the interaction between these two proteins appears limited (see Supplementary Figure S4 for additional information). To explore whether the interaction is an indicator for repair, we hypothesized that the PLA signal is correlated with proliferation rate, extent of inflammation, or p53 expression. Such signs of epithelial activation and stress may be accompanied by increased DNA damage (e.g., by oxidative stress). However, we could not detect any correlation with such features and PLA signal intensity. Although we could detect a reduction in PLA, MECP2 and XPC signal (Figure 7B–D) during progression from normal to cancer, we also observed a slight “recovery” of the PLA signal in the cancer samples (SCC in Figure 7B compared to high-grade dysplasias [HGD] lesions). This suggests a change in MECP2–XPC dynamics or a change in the frequency of the underlying cause for MECP2–XPC interactions.

In summary, the levels of XPC, MECP2, and XPC–MECP2 interactions decrease most drastically at the LGD-HGD transition.

## 3. Discussion

Comparing mammalian species of different maximum lifespans suggests widespread gene expression changes associated with pathways guarding tissue integrity and ensures cellular protection. Here, we have focused on one specific tissue type that is generally not represented in comparative analyses of mammalian biology [39,40,41,42,43,44], i.e., self-renewing squamous epithelia. Such epithelia are of special interest to the biology of longevity due to their relatively high proliferation and turn-over rate, their exposure to external stress factors, and their elevated age- and carcinogen-dependent risk for malignant transformation (e.g., most human cancers are carcinomas).

The mouse has served as the primary genetic model for studying squamous epithelia. However, it has become clear that the mouse shows “marked differences in the cellular heterogeneity” compared to humans [45]. Key differences are the quiescence of the human basal cell layer stem cells and the shift in proliferation into the parabasal cell population [5,25]. To better understand this peculiar human feature in the context of longevity, we have used an in situ proteomic approach.

### 3.1. The Meaning of Stem Cell Quiescence

Our theory that the observed slow-cycling/quiescent human basal cell layer is an adaptation to human longevity is supported by comparative studies of intestinal stem cells in naked mole rats [46]. In their words, “adaptations of adult stem cells…have one of the greatest impacts on organismal health and lifespan.” According to Montazid et al., adaptation to longevity in naked mole rats involves several modifications compared to mice: expansion of the size of the stem cell compartment, a slowdown in stem cell kinetics, and a turnover rate that more closely resembles humans rather than mice. Consequently, the reduced cell division rate of naked mole rat stem cells results in reduced somatic mutation rates [44]. In addition to the reduced cell divisions within the stem cell compartment, naked mole rats also show increased resistance to stress [47], a feature closely associated with longevity [48]. Therefore, the combination we have observed in human squamous epithelia stem cells of a reduced cell division rate and the increased expression of cytoprotective proteins that mediate stress-resistance [5] may form the backbone of adaptation to longevity and provides a flexible tool for evolution to modify cell behavior. This is in line with our observations of a spectrum of parabasal proliferation ranging from absent in mice to a hybrid form in monkeys, dolphins and dogs, and reaching its purest form in humans [25].

Another example of a species-specific difference in stem cell organization found in spermatogonial stem cell populations, where adult human (but not rodent) cells maintain a fetal-like signature—a feature the authors interpret as an adaptation to human longevity [49].

### 3.2. Alternative Explanations

Various explanations for the variable stem cell quiescence observed in different species that do not involve adaptation to longevity can be discussed; however, these alternatives lack the conclusiveness of the longevity adaptation hypothesis. The vast majority of the literature interprets stem cell quiescence as a protective mechanism to prevent cancer and tissue dysfunction [50,51]. Nonetheless, a quiescent stem cell layer may possess other features that could have driven its evolution, such as mechanical stress adaptation to help the oral epithelium withstand chewing certain foods. However, the general solution to such mechanical stress is typically keratinization and the formation of rete ridges, which are well-documented structural adaptations in various epithelial tissues, including oral epithelium, across mammals [52,53].

Alternatively, a quiescent stem cell layer could facilitate specialized niche signaling that is absent in most other species. However, there is no clear evidence that the niche environment of human squamous epithelium is fundamentally different from that in other mammals, nor that quiescent cells possess intrinsically different signaling functions compared to proliferating cells [54].

Human quiescent basal cells may also engage in specific immune system modulation, minimizing immune activation and chronic inflammation through specialized interactions with immune cells. Even if such an immune-modulatory function were unique to human quiescent basal cells, the resulting reduction in inflammation would inherently contribute to the longevity of the epithelium and, consequently, the organism. However, evidence supporting unique immune interactions specific to human quiescent stem cells especial squamous basal cells remains limited [55,56].

Another potential explanation is the protection against viral integration, particularly relevant in squamous epithelia of the Waldeyer’s ring area and the cervix. Low proliferation rates may reduce opportunities for viral integration and infection in basal cells, thereby decreasing the risk of developing squamous cell carcinomas. Nevertheless, viruses can integrate into both quiescent and proliferative cells, and other antiviral defense mechanisms exist that do not rely solely on reducing cell proliferation (one remotely relevant example: [57]).

Quiescence is often associated with a specific metabolic state [58]. One could argue that quiescent basal cells may have specialized metabolic profiles that support their long-term maintenance. However, long-term maintenance is intrinsically linked to longevity, suggesting that this adaptation ultimately supports our theory that the human basal cell layer represents an adaptation to longevity.

We have observed a distinct association of Polycomb proteins and other epigenetic regulators with basal and parabasal cells [25]. This could indicate that unique epigenetic landscapes in quiescent basal cells maintain their identity and functionality. However, epigenetic regulation is a common mechanism across various stem cell populations, regardless of their proliferation status, and does not inherently require quiescence. Thus, this too can be viewed as another facet of the longevity adaptation hypothesis.

Finally, one could argue that the observed stem cell quiescence in the basal cell layer enhances differentiation control, ensuring precise differentiation of stem cells and maintaining epithelial integrity and functionality. However, the exact mechanisms underlying this enhanced differentiation system remain unclear. Moreover, it is perplexing why such a system is not employed by other mammals, given their similar requirements for epithelial maintenance [59].

In summary, while alternative hypotheses offer potential explanations for the presence of a highly quiescent basal cell layer in human squamous epithelium, they either lack empirical support or overlap significantly with the primary longevity adaptation hypothesis. Therefore, the simplest and most straightforward explanation for the observed spectrum of basal quiescence and parabasal proliferation in mammals is an adaptation to longevity [25]. This corresponds well with the observation that lifetime stem cell divisions correlate with cancer risk [3]. Whether squamous epithelial stem cell quiescence is a uniquely human adaptation to longevity has to be seen. Recently Zhang and Gines stated that “it seems that every species studied so far has its unique pathway of cancer resistance because of different life histories and selective pressures” [60]. This suggest that studying species with vastly different longevity may produce valuable insights into how to protect humans better from cancer.

### 3.3. Proteomic In Situ Analysis Can Detect Putative Features of Longevity in Species Comparisons: XPC and MECP2

It has been speculated that one mechanism to enhance cell, particularly stem cell, cytoprotection is the enhanced expression of DNA repair genes. The elevated expression of DNA repair genes may contribute to increased longevity. Our analysis has focused on two genes associated with DNA repair, XPC [33] and MECP2 [34,37,61]. The expression of both proteins is elevated in human basal cells compared to epithelial cells in other mammals, especially rodents (ca. 5-11-fold up, Figure 1). Furthermore, the elevated expression of both proteins is associated with a specialized quiescent stem cell layer in humans, a cell layer that is absent in rodents. It is well documented that loss of XPC reduces life expectancy and causes skin cancers. Furthermore, in vitro XPC KO cells are highly sensitive to DNA damaging agents due to impaired DNA repair [38].

There is accumulating evidence that MECP2 plays a role in controlling oxidative stress and DNA damage [34,36,61,62,63,64,65]. This suggests that due to MECP2′s interaction with XPC and its specific location in epithelial stem cells, it could have cell protective functions.

It is interesting to note that the main type of mutation driving somatic mutation rates are C>T mutations due to deamination of 5′methylcytosine [44]. MECP2 is a protein that binds to sites of DNA methylation and CpG islands [66]. It is unclear whether MECP2 can protect against this specific mutation event, but a close relative, MBD4, has been suggested to function as a “safeguard against damage from 5mC deamination” [67]. However, MECP2 lacks the DNA glycosylase domain of MBD4 and therefore it is unclear how MECP2 may contribute to the protection of methylated CpG sites. Nevertheless, MECP2 has the ability to interact with a “mastermind” of DNA repair, XPC [37] while such interactions are not documented for MBD4. Whether this XPC–MECP2 interaction is crucial for maintaining stem cell DNA integrity remains to be seen. Interestingly, the loss of both MECP2 and XPC expression coincides with the formation of high-grade dysplastic (HGD) lesions (Figure 7C,D). This step coincides with the loss of the quiescent basal cell layer in precancer [68,69,70,71].

### 3.4. XPC-MECP2 Interactions Are Limited to Quiescent Basal Cells

Our in situ spatial analysis also allowed us to demonstrate a direct interaction between XPC and MECP2 that had been alluded to by previous studies [37]. Using a proximity ligation assay (PLA), the XPC–MECP2 interaction occurs specifically in quiescent basal cells. The number of PLA dots is relatively low in these basal cells, suggesting that only a small portion of MECP2 and XPC molecules interact. This interaction may represent a repair event which involves both MECP2 and XPC [37]. Since DNA damage occurs thousands of times per day per cell [72,73,74], MECP2 and XPC may provide a specific surveillance and repair system that is low in the non-stem cells of human squamous epithelia and is also low in rodent squamous epithelial cells. One of the functions of XPC is in Nucleotide Excision Repair (NER) in response to distortions in the DNA helix due to “bulky” DNA damage. Although it is difficult to estimate exact numbers, such DNA damage events involving XPC and NER may occur in the range of 10–10^2^ per day per cell, which could reflect the numbers of PLA dots we observed on 5 µm thick sections. Furthermore, XPC also participates in “smaller” DNA damage repair and can contribute to Base Excision Repair (BER; [75]). XPC may function as a “general sensor of damaged DNA” [76]. BER is likely to happen frequently in human cells and may not fit the low numbers of PLA signals per basal cell. On the other hand, basal cells have an elevated anti-oxidative damage program that could strongly reduce events requiring BER.

Alternatively, we observed PLA signals could represent random MECP2 and XPC interactions. If this were the case, we should expect a strict correlation between PLA signal and MECP2 and XPC expression. Although that is generally true, e.g., there is a drop in PLA signal from basal to suprabasal cells coinciding with a strong reduction in MECP2 and XPC expression (Figure 7). However, in SCC cells we observed a slight recovery of the PLA signal compared to high-grade dysplastic lesions (HGD, Figure 7B). This suggests that even in SCC cells with lower MECP2 and XPC expression, interactions between these two proteins still manifest. Our hypothesis is that this increase in interaction is specifically due to increased DNA damage burden in SCC cells. In summary, we observe a reduction in PLA signal associated with progression towards cancer. Whether this loss is associated with the loss of quiescent stem cell features or whether it is just due to the loss of MECP2 and XPC expression levels remains to be shown. Further investigations are required to dissect the significance of MECP2 and XPC interactions and how they change during progression towards cancer.

### 3.5. Enriched Stem Cell Pathways in Human Squamous Epithelia

To address what may make the human basal cell layer special beyond low proliferation rates, we performed comparisons between existing scRNAseq datasets from mice and humans. As summarized in Table 2, the markers present in at least two human scRNAseq datasets and in our proteomic dataset, can be interpreted as indicators of readiness, such as metabolic changes, infection, and wounding. Consistent with basal cells being attached to the basal lamina, one subset of markers is associated with this “household” function.

For example, ASS1, a critical enzyme in the urea cycle, is a component of the quiescent basal cell proteome difficult to comprehend. However, most non-hepatic cells do not express most urea cycle enzymes. For example, there is no indication for OTC and ARG1 gene expression in quiescent basal cells of squamous epithelia. High ASS1 levels may provide basal cells high flexibility in case of arginine deprivation and usage of citrulline. What conditions may require ASS1 activity in squamous basal cells is unknown. This example highlights that several basal cell markers have no clear-cut function unless stress and emergency conditions are considered.

### 3.6. A Combo of Protective Features to Enhance Stem Cell Resilience?

Beyond DNA repair related genes (e.g., the aforementioned XPC, MECP2 and RAD23B, PML, DDB2), our analysis suggests the existence of a set of human specific (absent in mouse) basal stem cell markers that also includes NRF2-related anti-ferroptosis genes (ABCC3, CBR1, SLC6A15, GSR, SLC39A13, SLC2A5, PRNP, SLC40A1), and defense genes (e.g., IFITM3, CD74, CD40; IFITM2, ERAP2, IL18, IFIT3, PML, GHR, BST2).

This elevated emphasis on cytoprotection in a human stem cell population may be the foundation of low mutation rates per bp per year in humans in general. For example, humans have the lowest substitution rates per genome per year in a comparative study on colon samples [44]. How the somatic mutation rate per year is reduced almost 17-times in humans compared to mice is unclear [44]. We know little about the stem cell organization in the colon in different species. Whether there is a dedicated quiescent stem cell population in human colonic crypts that is absent in other species, is also unknown. The authors concluded that in the colon, the mutations are driven by, “endogenous mutational processes … including 5-methylcytosine deamination and oxidative damage” [44] and that, “cell division rates are not a major determinant of somatic mutation rates across human tissues.” However, this assumption that the somatic mutation rate is independent of the number of cell divisions is misleading and the data of Abascal et al. [77] actually show a 2-3-fold variation in mutation rates and the highest mutation rate in the cell type with the highest proliferation rate (i.e., the colon). The idea that “modest” differences in mutation rate due to differences in cell division numbers are irrelevant to the risk of cancer, appears hard to sustain. A two-fold increase in mutation rate may make certain tissues the “weakest link” and cause problems in the long run. For example, the mere 2-3-fold increase in mutation rates increases the cancer risk of patients with MUTYH [78], and 1.2-7-fold for POLE and POLD1 mutations [79]. Surprisingly, the increased mutation rate in patients with germline mutations in these genes do *not* show overt premature aging phenotypes. Furthermore, mutational burden is associated with origins of replication [80,81].

In this context, we also would like to highlight that the cytoprotective quiescent state of the basal stem cells is extremely stable and only broken in extreme conditions [5]. Recently, studies on inflammatory states have confirmed this robustness of “deep” quiescence in the human esophagus by Rochman et al. observing that inflammation leads to “hyperproliferation of less mature epithelial subpopulations yet stability of the quiescent, stem cell–enriched basal epithelial compartment” [82].

### 3.7. Limitations of the Study

While we have discussed several explanations for the presence of the human basal quiescent stem cell layer (see Section 3.1 and Section 3.2), we cannot rule out that factors unrelated to longevity adaptation have influenced the development of this anatomical feature. A key limitation is that our study did not cover all mammalian species. Out of approximately 6400 known living mammalian species, we investigated only 16—omitting many species noted for extreme longevity. However, we did include several rodents (including mice), and our longevity distribution roughly matches that observed across all mammals (see bottom of Supplementary Table S1).

Consequently, determining how “unique” human stem cell organization is remains challenging. Interestingly, we observed no obvious differences in stem cell proliferation and basal cell organization between long-lived rodents—such as *Spalax ehrenberg* and *Sciurus carolinensis* (each with a maximum lifespan above 20 years)—and shorter-lived species like mice and rats (here and [25]). This suggests that adaptation to longevity is not a one-size-fits-all process.

Additionally, our study examined how the progression from normal tissue to dysplasia to cancer impacts basal stem cell layer quiescence, and we explored the point at which normal stem cell behavior might be lost during human squamous cell carcinogenesis. However, we do not yet understand whether or how normal stem cells transform into cancer stem cells (CSCs). Our data indicate that a critical step toward the loss of normal stemness and basal cell quiescence likely occurs in high-grade dysplasias (HGDs)—a pathological state associated with the disruption of a normal basal cell phenotype [68,69,70,71]. This change may represent an important step toward the elusive acquisition of invasive behavior, a molecular event that remains surprisingly poorly understood [83].

## 4. Materials and Methods

### 4.1. Antibody List

See Supplementary Tables S1, S3, S5, and S8.

### 4.2. Antibody Evaluation for Species

To determine whether an antibody “works” to detect its target epitope in a specific species, we used the following criteria: 1. The staining patterns are as expected and reflects human protein expression patterns based on the literature, our experience, and the Human Protein Atlas, 2. If information on the epitope is available, we compared such protein sequence data from different species, 3. Checking antibody supplier data for their recommended use for which species, 4. Exploring the publication record for the antibody and its use in different species. These data are documented in Supplementary Table S1.

### 4.3. Manual Multiplex Cyclic Immunofluorescence (mcyIF) Stainings

5 µM formalin-fixed, paraffin-embedded (FFPE) tissue sections were deparaffinized with xylenes, rehydrated, and antigen retrieval was achieved by incubating for 13 min in 1xTE in a Cuisinart pressure cooker [84,85] and remaining in the pressure cooker for another 10 min. After removing from the pressure cooker, slides were cooled down for 20 min and incubated simultaneously with two to three primary antibodies that are directly conjugated with AlexaFluor488 or FITC, AlexaFluor555 or PE, and AlexaFluor647 or APC. Incubation occurred overnight at room temperature in 1xPBS. For antibodies that were not directly conjugated, two primaries were added, one made in rabbits and one in mice. In that case, after washing with 1xPBS, secondary antibodies were applied and incubated for 15 min: a mix of for example: horse Anti-Rabbit IgG Antibody (H+L), DyLight^®^ 594 (DI-1094, Vector Laboratories, Newark, CA, USA) and horse Anti-Mouse IgG Antibody (H+L), DyLight^®^ 488 (DI-2488, Vector Laboratories, Newark, CA, USA), both 1:400 diluted in 1xPBS with 5%BSA. After washing with 1xPBS, sections were counterstained with 2µM Hoechst dye for 10 min. After washing, slides were provisionally mounted with VECTASHIELD^®^ Antifade Mounting Medium (H-1000, Vector Laboratories, Newark, CA, USA) and imaged using a Leica SP5 confocal microscope (Leica Microsystems, Deerfield, IL, USA) with a motorized stage and the 63× (Leica 1.4-0.6 HCX PL APO, oil) or 20x objective (Leica 20×/0.7 HC PL APO). After imaging, slides were unmounted and washed in 1xPBS. Antibodies were removed by microwaving slides in 1xTE buffer. Boiling the slides was repeated five more times over the next 40 min and then the slides were cooled down to room temperature. Additionally, a peroxide-light bleaching step was included especially for stains using directly conjugated antibodies: sections were incubated between 10 and 45 min in a mix of 7.5 mL PBS, 0.24 mL 1M NaOH, 1.35 mL 30%H_2_O_2_ and exposed to maximum light intensity from two Aliyeah Xz-06 22600Lux LED light sources. Photobleaching times were dependent on signal intensity and fluorochrome. Then, the next round of primary antibodies was applied. The same areas from the initial imaging session were imaged, analyzed, and quantified using Leica LASX software version 3.7 (Leica Microsystems, Deerfield, IL, USA). To produce overlay images from all the cycles of the same tissue area, Leica files were exported as TIFFs and assembled in Adobe Photoshop version 26 (Adobe, San Jose, CA, USA). Images were analyzed using Miltenyi MACSiQView 1.2.2 and 1.3 software (Miltenyi Biotec, Gaithersburg, MD, USA). In manual analysis, we relied on visual determination of basal cell identity due to high resolution and high magnification of images. Additionally, we used basal cell markers as guides of basal cell identity. In Figure 2, we used this “manual” determination of basalness to compare basal and suparabasal gene expression. For scatterplots, we determined the position of the gates by determining the lowest level of expression of clearly positive cells within the images.

The manually multiplex images were also used to determine the gene expression and number of delaminating cells. A delaminating cell was defined as a cell that has still a connection to the basal membrane but has left the group of basal cells and moved upwards out of the basal plain.

### 4.4. Automated Multiplex Immunofluorescence Staining Using the Miltenyi MACSima Platform

For antigen retrieval, tissue slides were incubated for 20 min at 98 °C in 1xTEC pH9 buffer (made from 10x TEC: 2.5 g Trizma Base (Sigma-Aldrich/Merck, Burlington, MD, USA, # 93362), 5.75 g EDTA Disodium salt dihydrate (Sigma-Aldrich/Merck, Burlington, MD, USA, #E5134), 3.2 g Sodium citrate tribasic dihydrate (Sigma-Aldrich/Merck, Burlington, MD, USA, #1064480500), pH adjusted with NaOH. After cooling down, the slides were mounted onto a MACSwell Imaging Frame and loaded onto the MACSima staining and imaging platform (all Miltenyi Biotec, Gaithersburg, MD, USA). Following the manufacturer’s recommendations, Miltenyi antibodies were diluted in MACSima Running buffer. After preprocessing of image data, images were analyzed using Miltenyi MACSiQView 1.2.2 and 1.3 software (Miltenyi Biotec, Gaithersburg, MD, USA).

### 4.5. Proximity Ligation Assays (PLA)

The PLA was performed on a tissue microarray (TMA) slide (BBS02011 from TissueArray.Com LLC, Derwood, MD, USA using the Duolink^®^ Proximity Ligation Assay from MilliporeSigma, Burlington, MA, USA according to the manufacturer’s recommendations with the following changes: Primary antibodies (MECP2 1:100, clone D4F3, #3456, Cell Signaling Technology, Danvers, MA, USA) and XPC 1:100; D-10, sc-74410, Santa Cruz Biotechnology, Dallas, TX, USA) were incubated overnight at room temperature. Secondary antibodies (Duolink in situ PLA probe anti rabbit plus and Duolink in situ PLA probe anti mouse minus) were incubated for 2 h at 37 °C. The ligation reaction was extended to 1 h and the amplification reaction to 110 min. The TMA slide was imaged using a Leica Sp5 confocal microscope using a 20× objective. After imaging, the cover glass was removed in water and the TMA incubated with anti-rabbit IgG Alexa647 (A21245 Invitrogen, Carlsbad, CA, USA) and anti-mouse IgG Fluorescein (FI-2000 Vector Laboratories, Newark, CA, USA) to detect and visualize MECP2 and XPC, respectively. After overnight incubation, the TMA was imaged again at the same location that the PLA signal was imaged. After washing off the cover glass and photobleaching the exiting fluorescent signals, a KRT10/13 antibody (clone REA1138 FITC Miltenyi Biotec, Gaithersburg, MD, USA) and a Ki-67 antibody (clone D3B5 AlexaFluor647, Cell Signaling Technology, Danvers, MA, USA) were added overnight and imaged again the next day to aid in cell clustering in downstream UMAP analyses. After cooking the slide in the microwave and photobleaching for 25 min, the TMA slide was incubated one last time with KRT14 (REA1145 FITC Miltenyi Biotec, Gaithersburg, MD, USA), VIM (D21H3 PE Cell Signaling Technology, Danvers, MA, USA), and SLC3A2 (e-5 sc376815 AlexaFluor647, Santa Cruz Biotechnology, Dallas, TX, USA). The images were aligned in Photoshop using the DAPI stains of each day. The resulting TIFF images were imported into MACSiQView version 1.3 (Miltenyi Biotec, Gaithersburg, MD, USA) for further analysis and quantification. Additional PLA controls were performed ahead of the TMA stain using two Ki-67 antibodies as positive control (see Supplementary Figure S4). After the end of staining and imaging, a standard hematoxylin and eosin stain was performed and imaged.

### 4.6. Tissues

Animal tissues and their collection have previously been described [25]. Human tissue samples were obtained from the Cooperative Human Tissue Network (CHTN). Additional human samples were obtained from TissueArray.Com LLC, Derwood, MD, USA in form of human tissue microarrays (TMAs: ES809, BBS02011).

### 4.7. Analysis of scRNAseq Data

Datasets included in the study are from published work [82,86,87,88,89,90,91,92]. For the gene sets specifically expressed, see Supplementary Table S6.

### 4.8. Statistical Analysis

Statistical analyses and graphs were generated using Prism Software (version 10.2.3, Graphpad, Boston, MA, USA). Statistical significance was determined using Student's t-test, with *p*-values ≤ 0.05 denoted by an asterisk (*). For Figure 1, Figure 3, and Figure 5, data are represented as the median with the 25th and 75th percentiles (interquartile range, IQR), and the whiskers extend from the minimum to the maximum values. In Figure 7, outliers are shown; here, the whiskers extend to the minimum and maximum values within 1.5 times the IQR, with outliers plotted as individual points.

## Figures and Tables

**Table 1:** Proliferation state of delaminating cells: Using proliferation markers MCM2, PCNA, phospho-histone H3 (PHH3).

Species	MCM2 %	PCNA %	PHH3 %	Ki67 %
Human	40	40	0	20
Dog	100	N/A	N/A	100
Monkey	50	N/A	N/A	N/A
Mouse	N/A	100	0	40
Rat	100	100	0	50

**Table 2:** Selected high-quality basal cell markers and their putative stem cell functions.

Basal Cell Marker	Function	Putative Role in Basal Cells	Related Basal Proteins
ABCC3	cellular waste export of often GSH conjugated molecules; detoxification	enhanced protection against cytotoxic substances.	GSR, GSS
ASS1	urea cycle; arginine synthesis	Without other enzymes of the urea cycle, ASS1 cannot make arginine. However, NOS1 could make arginine into citrulline and ASS1 could use this as substrate to start making arginine again.	SLC3A2
CAV1	scaffolding protein of caveolae plasma membranes; regulatory function of endocytosis, antiproliferative	enhance adhesion to basal lamina; may sequester growth factor receptors and deepen quiescence; in caveolae: absorbing mechanical stress and preventing cell damage (membrane reservoir); may contribute to deep quiescence due to its antiproliferative effects; differentiation inhibitor; may have link to ferroptosis.	CAV2, CAVIN1, FLOT1, BSG, DST, ITGB1, ITGA6, LIMA1, NGFR, SLC1A3, SLC3A2, SLC7A5, CDH13
CAVIN1	scaffolding protein of caveolae plasma membranes	enhance adhesion to basal lamina; may sequester growth factor receptors and deepen quiescence; in caveolae: absorbing mechanical stress and preventing cell damage.	CAV1, CAV2, FLOT1
CDH13	cell membrane and is anchored by a GPI moiety, rather than by a transmembrane domain	may function as an adiponectin receptor? Tumor suppressor?	ITGB1
COL17A1	attachment of basal keratinocytes to the underlying basement membrane	“household” protein to adhere basal cells to the basal lamina.	DST, LAMC1, ITGB1, ITGB4,
CXCL14	chemoattractant for immune cells; may mediate stromal reprogramming	may mediate antimicrobial activity; problem: how is it kept in an inactive state before needed? Main target may be fibroblasts which express high levels of the putative CXCL14 receptors LRP1 or GPR85. May also be required for the recruitment of dendritic cells and macrophages to sites of infection or inflammation but CXCL4 ko mice have no phenotype; potential anti-tumor activity. Enigma.	
CYBRD1	Iron metabolism, iron uptake	Ferroptosis? Iron metabolism related to FTH1/FTL: reduces iron for storage by ferritin?	FLOT1, LIMA1, FTH1
DKK3		only receptor for DKK3 is CKAP4. CKAP4 mainly expressed on differentiated keratinocytes, fibroblasts.	
DST	hemidesmosome linker to cytoskeleton	“household” protein to adhere basal cells to the basal lamina	COL17A1, KRT14
FAT1	cell–cell adhesion, cell polarity, WNT and Hippo signaling	tumor suppressor function may provide a safety feature for stem cells.	LATS1, YAP1
FBXO2	binds denatured glycoproteins, preferentially those of the high-mannose type	Defense: detection of bacterial surface sugars and viral glycoproteins? Maintenance: Clearance of damaged lysosomes?	
FHL2	scaffolding protein, interaction with dozens of proteins,	maybe advantageous to maintain ER health? Enigma.	
IFITM3	prevents the fusion of viral membranes with host cell membranes	without infection: maintain membrane stability and integrity, Readiness for viral infection and turning of interferon response. Elevated level of readiness. Saves time to reach high enough IFITM3 levels to prevent viral entry.	IFITM1, IFITM2, TLR3, CAV1,
IL18	cytokine	may be “stored” as precursor pro-IL18 to make the cell ready for an emergency and accelerate response times; hypothesis: IL18 exists in its pro-form (supported by PMID: 37068092).	IFITM1, IFITM2, IFITM3
IL1R2	decoy receptor for IL1	Dampens inflammatory signaling; maybe protects basal stem cells from activation by “low” level inflammatory signals and maintains a quiescent state even in inflammation (see PMID: 38637492).	
ITGB4	hemidesmosomes	“household” protein to adhere basal cells to the basal lamina.	ITGA6, LAMB2, LAMB3, LAMC1, LAMC2; COL17A1, DST, KRT14
KRT14	Intermediate filament cytoskeleton	“household” protein to adhere basal cells to the basal lamina.	ITGA6, LAMB2, LAMB3, LAMC1, LAMC2; COL17A1, DST, KRT14
KRT19	Intermediate filament cytoskeleton	“household” protein to adhere basal cells to the basal lamina.	
LAMB3	basal lamina	“household” protein to adhere basal cells to the basal lamina.	
MOXD1	unknown	Enigma.	
MXRA5	TGF-β1-regulated with anti-inflammatory and anti-fibrotic properties		
NFIB	Transcriptional regulator	Inhibits EZH2, a marker of parabasal cells. May “govern super-enhancer maintenance” specific for *quiescent* basal stem cells.	SOX2, SOX6
NINJI1	anti-bacterial host defense by plasma membrane rupture	May supercharge inflammation in case of catastrophic infection of stem cells.	
NTRK2	BDNF receptor	Enigma.	DST, NGFR
PCDH7	cell–cell-adhesion, RHO signaling		MECP2, AHRGEF28
SH3PXD2A	scaffolding protein prepares a cell for migration, invasion	potentially provides readiness for cell migration.	FSCN1, LIMA1
SLC1A3	high affinity glutamate transporter	glutamate can be a substrate to make other amino acids or made into glutamine for ammonia detoxification by GLUL.	GLUL
SLC1A4	neutral amino-acid transporter that mediates transport of alanine, serine, cysteine, proline, hydroxyproline and threonine.	Cysteine uptake for GSH production.	GSR, GSS
SLC7A5	uptake of large neutral amino acids	may import amino acids and activate mTOR pathway.	SLC3A2
SOX6	transcription factor	growth inhibitor; gene targets unknown that may mediate quiescence; may regulate extracellular matrix gene expression; unknown partner in quiescent basal cells.	BCL7A, FLOT1, NFIB, NFIC, SOX2, ZNF503
TNS1	cell–matrix adhesion	“household” protein to adhere basal cells to the basal lamina.	ITGB1
SUMMARY	1. Household function in cell–matrix adhesion. 2. Cell protection.3. Many genes that by themselves appear “useless”.	Explanations: 1. readiness for emergencies regarding wounding and infection, nutrient deficiencies; 2. feeder cell function for suprabasal cells (large set of transporter molecules); 3. enhances cellular protection.	IL18, CXCL14, IFITM3, GSR, GSS, SLC3A2, SLC7A5, SLC7A8, SLC1A3, ASS1

## Data Availability

Upon request, we will share the protein expression data from our multiplex expression analyses in form of Excel sheets, .csv or .fcs files.

## Supplementary Materials

The following supporting information can be downloaded at: https://www.mdpi.com/article/10.3390/ijms26073144/s1.
