# Supplementary material for: Human-Specific Organization of Proliferation and Stemness in Squamous Epithelia: A Comparative Study to Elucidate Differences in Stem Cell Organization
Source: Int J Mol Sci. 2025 Mar 28;26(7):3144. doi: 10.3390/ijms26073144 (PMC11989042; doi:10.3390/ijms26073144)
Supplement: Supplementary file 1 [file ijms-26-03144-s001.zip › Supplementary Figure S1 XP and MDP.pdf]

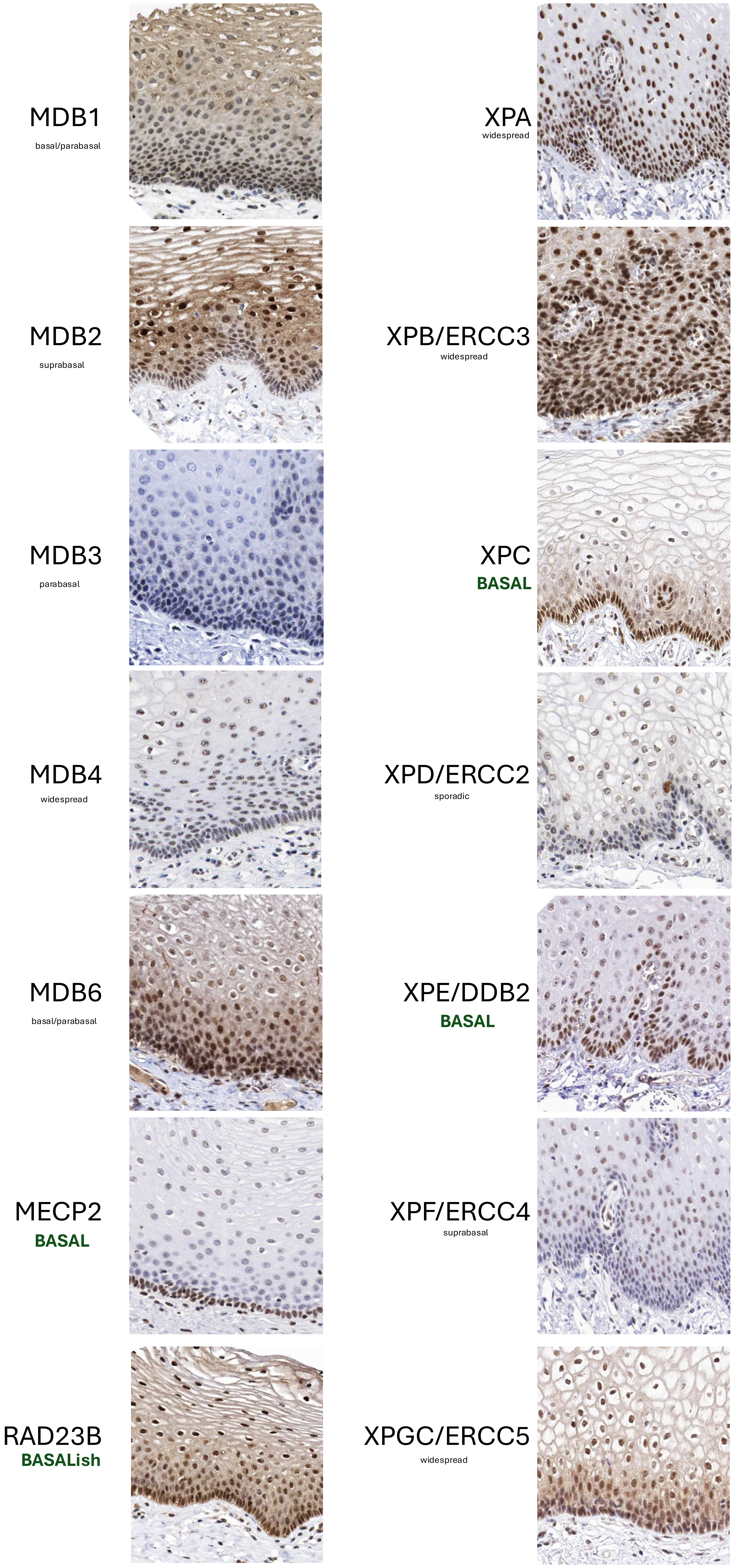
MDB1
basal/parabasal
XPA
widespread
MDB2
suprabasal
XPB/ERCC3
widespread
MDB3
parabasal
XPC
BASAL
MDB4
widespread
XPD/ERCC2
sporadic
MDB6
basal/parabasal
XPE/DDB2
BASAL
MECP2
BASAL
XPF/ERCC4
suprabasal
RAD23B
BASALish
XPGC/ERCC5
widespread
