# Supplementary material for: Human-Specific Organization of Proliferation and Stemness in Squamous Epithelia: A Comparative Study to Elucidate Differences in Stem Cell Organization
Source: Int J Mol Sci. 2025 Mar 28;26(7):3144. doi: 10.3390/ijms26073144 (PMC11989042; doi:10.3390/ijms26073144)

Supplementary Table S2.

Markers	Raw values								Histone H1 normalized values								Stroma normalized values								Expression Pattern
	Hs		Mk		R		Ms		Hs		Mk		R		Ms		Hs		Mk		R		Ms		
**Cell Cycle**	B	Sb	B	Sb	B	Sb	B	Sb	B	Sb	B	Sb	B	Sb	B	Sb	B	Sb	B	Sb	B	Sb	B	Sb	
MCM2	2.75	9.97	16.99	6.40	24.45	12.37	14.42	3.26	0.07	0.37	0.55	0.52	3.07	1.56	1.19	0.84	0.17	0.58	1.57	0.80	1.68	1.26	1.91	0.41	Hs: Sb; Mk/R/Ms: B
**Stem & Basal Cell Markers**																									
TP63	70.44	34.85	16.85	8.42	20.40	13.75	47.60	26.22	1.67	1.26	0.53	0.75	2.58	1.89	2.13	1.28	N/A	N/A	N/A	N/A	N/A	N/A	N/A	N/A	Hs/Mk/R/Ms: Nuclear
MECP2	35.88	4.17	28.70	14.93	4.30	6.61	10.02	11.96	1.04	0.15	0.74	0.45	0.49	0.90	0.48	0.60	0.99	0.06	0.95	0.59	0.21	0.26	0.20	0.24	Hs/Mk: B; R/Ms: All, reduced
XPC	44.41	7.55	18.75	14.29	4.12	3.56	5.90	5.79	1.37	0.40	1.53	0.89	0.50	0.48	0.27	0.28	2.35	0.42	1.44	1.04	0.57	0.51	0.21	0.14	Hs/Mk: B; R/Ms: Reduced
**Normalizer**																									
Histone H1	43.11	29.90	23.01	24.74	10.94	9.24	26.16	22.82	1.00	1.00	1.00	1.00	1.00	1.00	1.00	1.00	1.08	1.06	1.89	1.86	0.68	0.62	0.97	0.98	Hs/Mk/R/Ms: Nuclear; All cells

Hs, Homo sapiens; Mk, monkey; R, rat; Ms, mouse. Sb, suprabasal; B, basal.

Human vs mouse

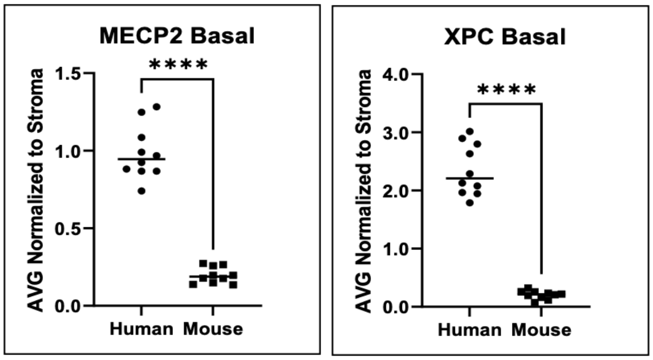

human

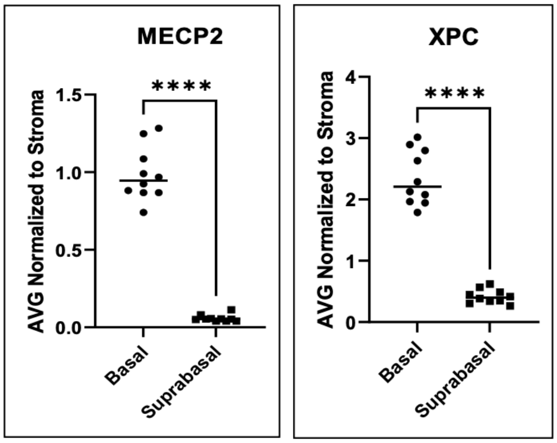

human

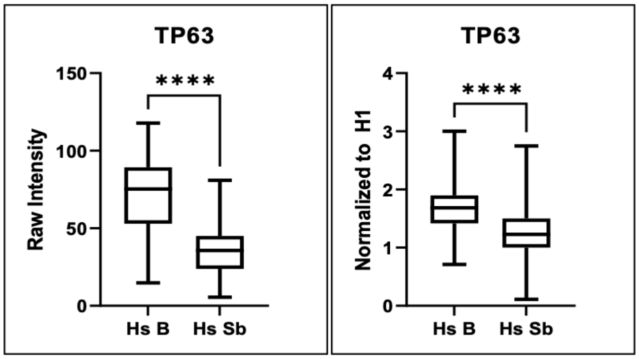

Supplement: Supplementary file 1 [file ijms-26-03144-s001.zip › Supplementary Table S2 basal celle xpression in different species.pdf]
