# Supplementary material for: Human-Specific Organization of Proliferation and Stemness in Squamous Epithelia: A Comparative Study to Elucidate Differences in Stem Cell Organization
Source: Int J Mol Sci. 2025 Mar 28;26(7):3144. doi: 10.3390/ijms26073144 (PMC11989042; doi:10.3390/ijms26073144)
Supplement: Supplementary file 1 [file ijms-26-03144-s001.zip › Supplementary Table S7 ferroptosis HPA.pdf]

Table S7.

**gene name**	HPA	Ferroptosis (A=anti-ferroptotic; P=pro-ferroptotic)	HPA interpretation	Basal human specific supplementary Table T5	Function	NFE2L2 regulated	PUBMED ferroptosis 5755 articles
**EMC2**	yes	P	basal				3
**NCOA4**	yes	P	basal				112
**SLC1A5**	yes	P	basal and parabasal				22
**CHAC1**	yes	P	no clear pattern				32
**STEAP3**	yes	P	no clear pattern				21
**BECN1**	yes	P	no clear pattern				29
**CARS1**	yes	P	no clear pattern				6
**IREB2**	yes	P	no clear pattern				14
**RPL8**	yes	P	no clear pattern				8
**ATG5**	yes	P	no expression				31
**TF**	yes	P	no expression				207
**SLC38A1**	yes	P	no expression				9
**ALOX15**	yes	P	no expression				34
**ALOX5**	yes	P	no expression				24
**ALOX12**	yes	P	suprabasal				19
**BACH1**	yes	P	suprabasal				14
**GLS2**	yes	P	suprabasal				16
**PGD**	yes	P	suprabasal				8
**TFRC**	yes	P	suprabasal		iron import		79
**G6PD**	yes	P	suprabasal trend				24
**CAV1**	yes	A	basal				21
**CISD1**	yes	A	basal				22
**FTH1**	yes	A	basal		iron storage	yes	147
**FTL**	yes	A	basal	yes	iron storage	yes	34
**GCLC**	yes	A	basal	yes		yes	23 (glutathione 1599)
**GSR**	yes	A	basal	yes		yes	5
**GSS**	yes	A	basal			yes	14 (glutathione 1599)
**OTUB1**	yes	A	basal				11

**SLC3A2**	yes	A	basal		cystine import		36 (CD98 13)
**CISD2**	yes	A	basal trend				8
**DHODH**	yes	A	basal trend				17
**GPX4**	yes	A	basal trend		ROS detox	yes	1373
**NFS1**	yes	A	basal trend				9
**HSPA5**	yes	A	basal and parabasal				18
**CBS**	yes	A	no expression				18
**SLC40A1**	yes	A	no expression				17
**AKR1C2**	yes	A	parabasal			yes	4
**AKR1C3**	yes	A	parabasal	yes		yes	11
**FANCD2**	yes	A	parabasal				32
**NQO1**	yes	A	parabasal			yes	33
**AKR1C1**	yes	A	suprabasal	yes		yes	14
**FDFT1**	yes	A	suprabasal				6
**ATP5MC3**	no	P					6
**LPCAT3**	no	P					25
**NOX1**	no	P					23
**NOX4**	no	P					32
**PHKG2**	no	P					7
**AIFM2**	no	A					23
**FTMT**	no	A					7
**MT1G**	no	A				yes	12
**SLC7A11**	no	A		yes			517
