# Supplementary figures and images for: Human-Specific Organization of Proliferation and Stemness in Squamous Epithelia: A Comparative Study to Elucidate Differences in Stem Cell Organization
Source: Int J Mol Sci. 2025 Mar 28;26(7):3144. doi: 10.3390/ijms26073144 (PMC11989042; doi:10.3390/ijms26073144)

Primarily basal

suprabasal

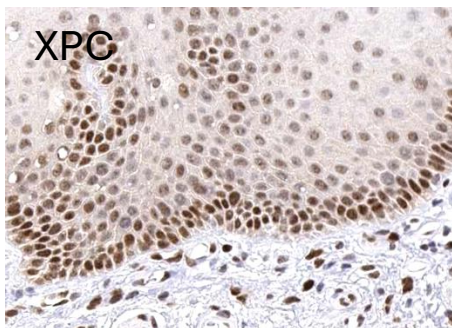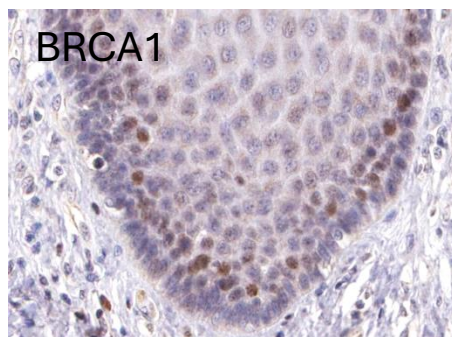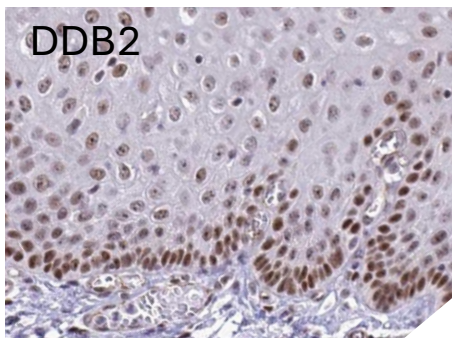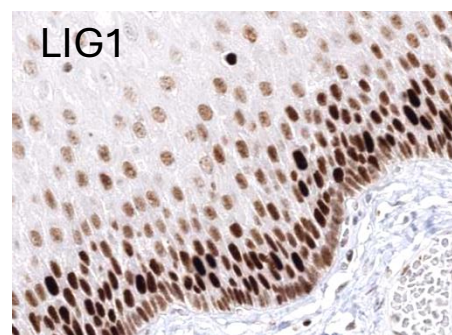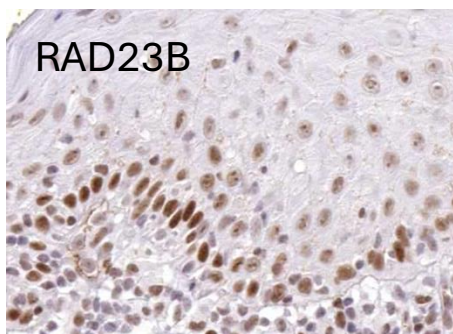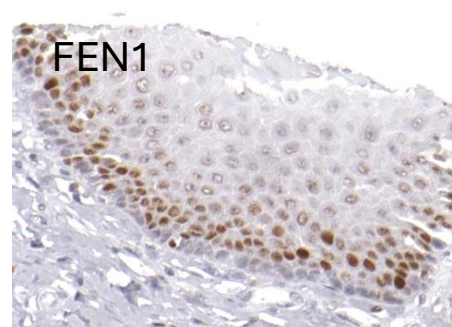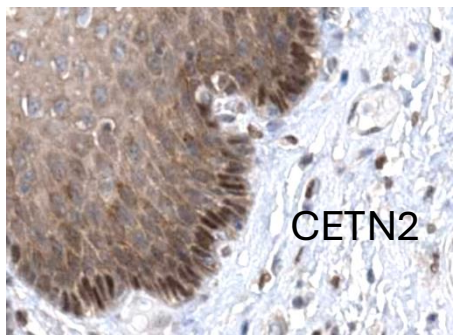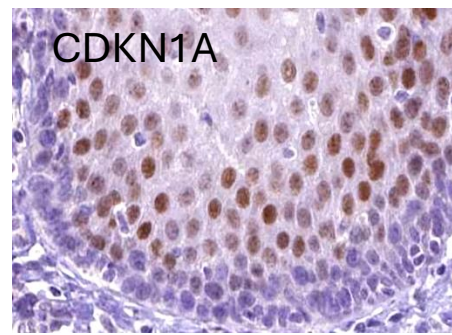

Supplement: Supplementary file 1 [file ijms-26-03144-s001.zip › Supplementary Figure S2 DNA damage.pdf]

FTH1

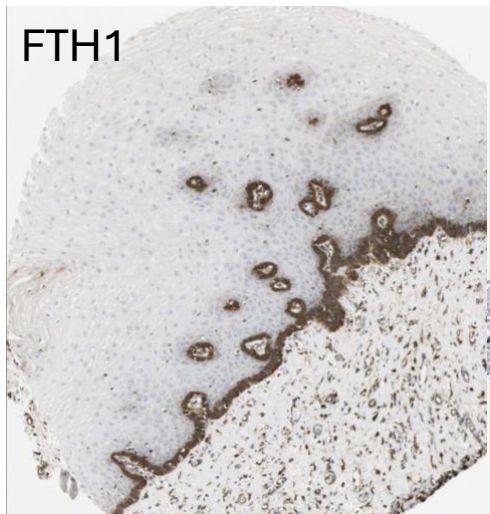

FTL

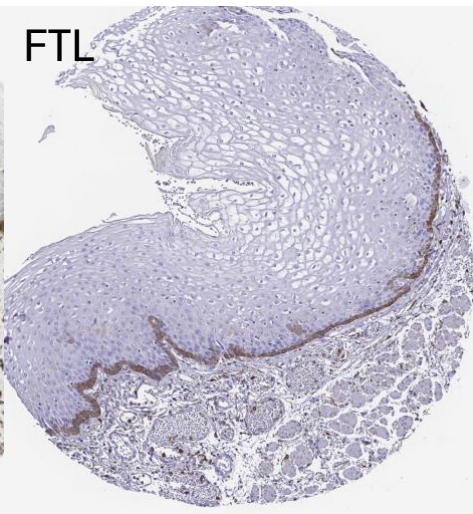

SLC3A2

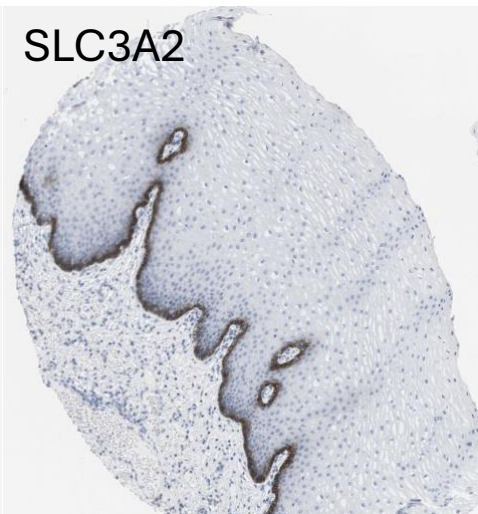

GSR

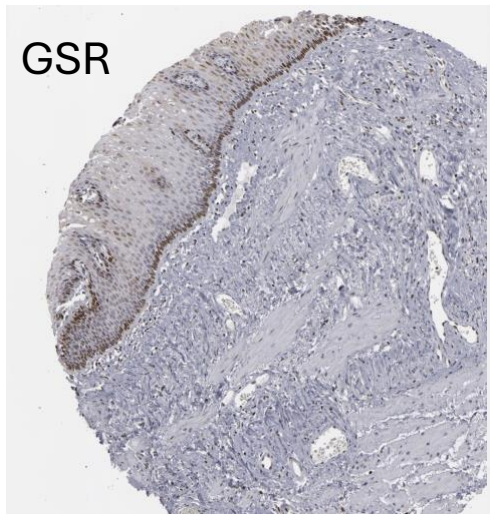

GCLC

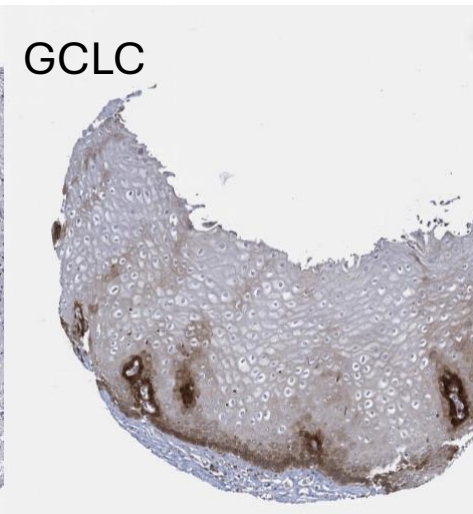

GSS

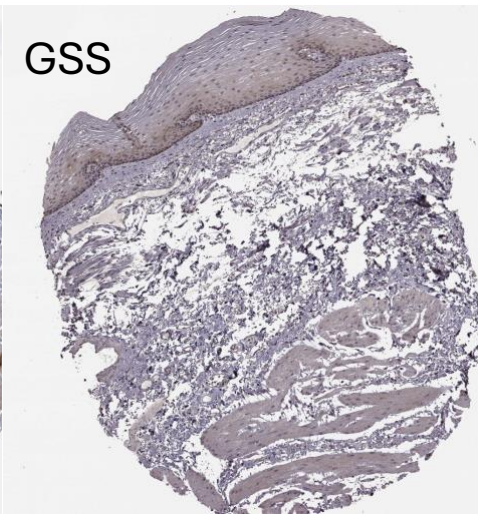

CAV1

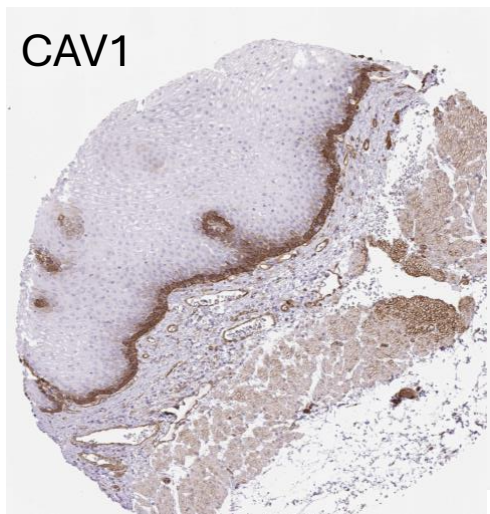

CISD1

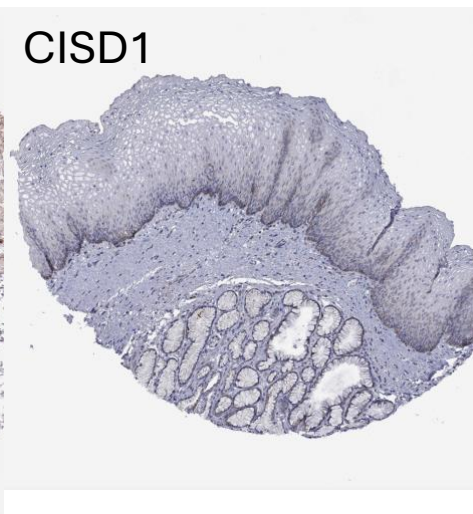

OTUB1

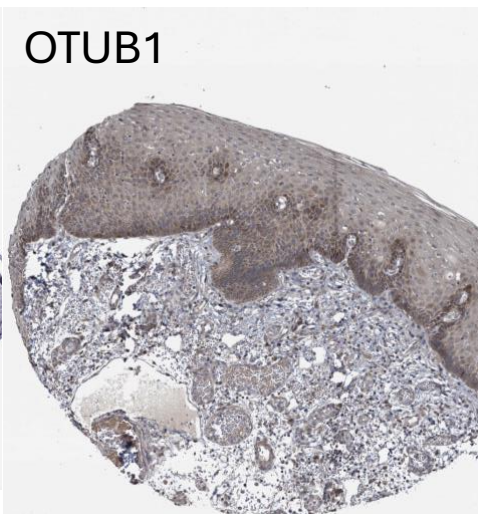

Supplement: Supplementary file 1 [file ijms-26-03144-s001.zip › Supplementary Figure S3 ferroptosis markers.pdf]

A

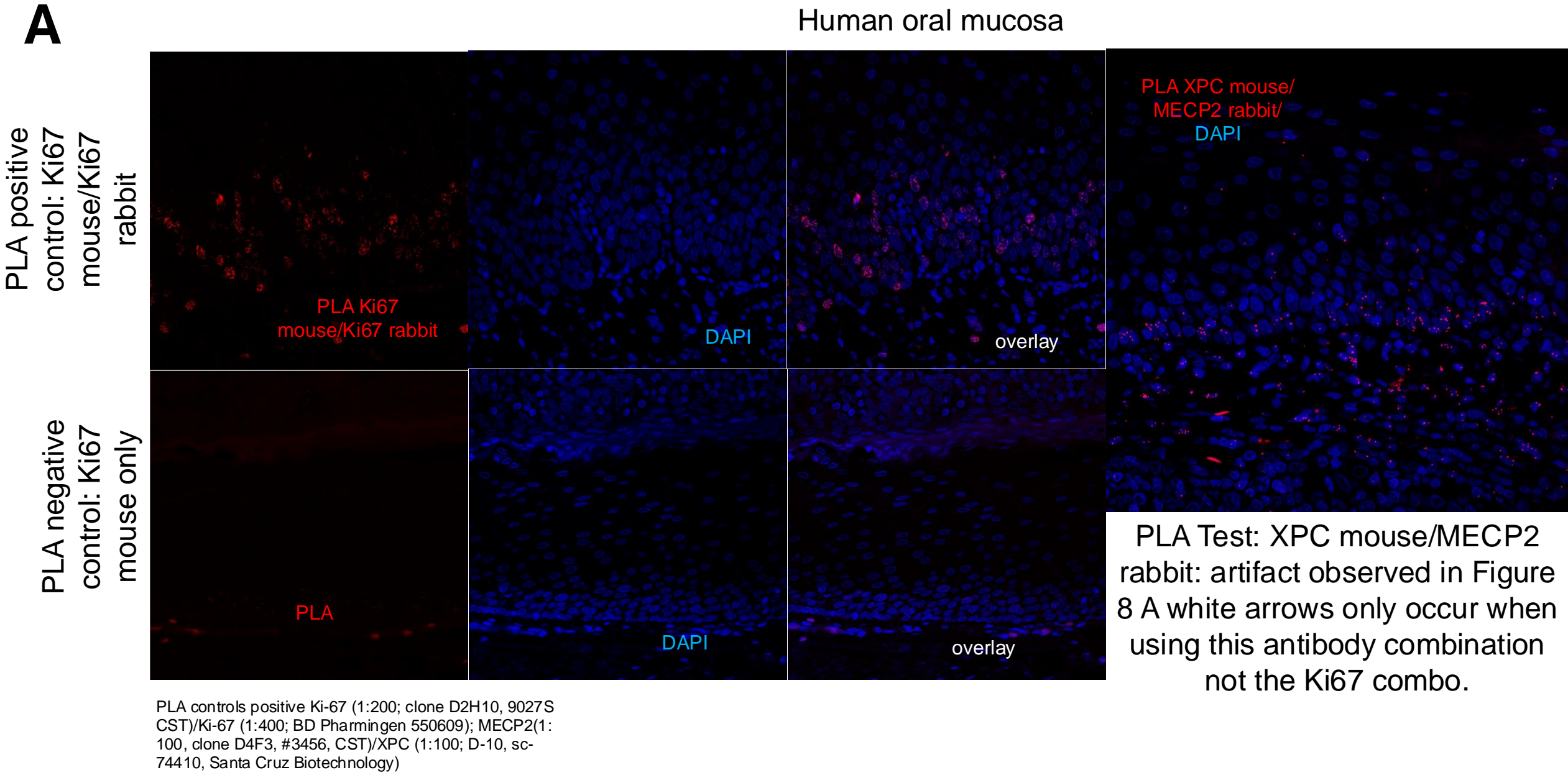

B

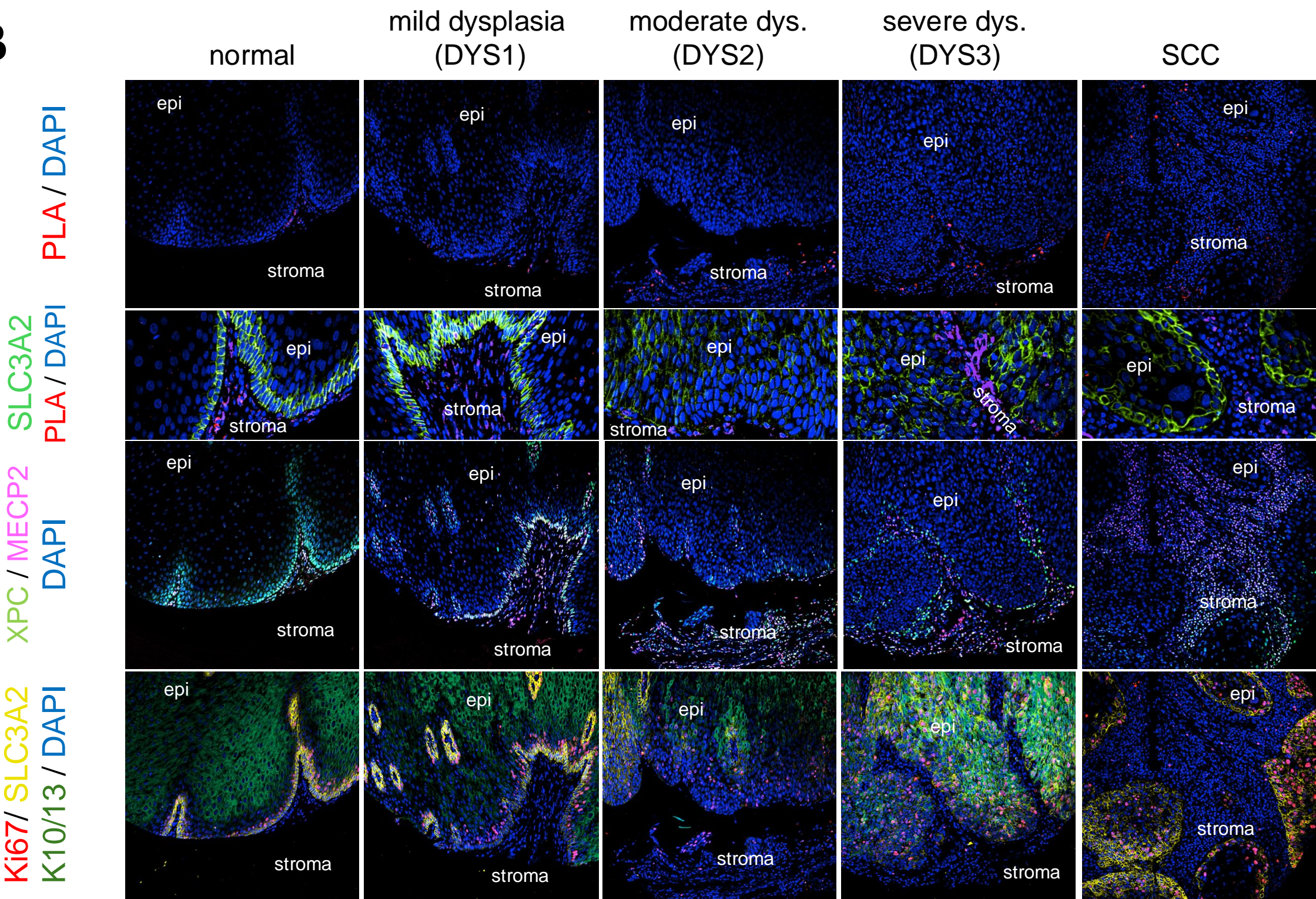

C

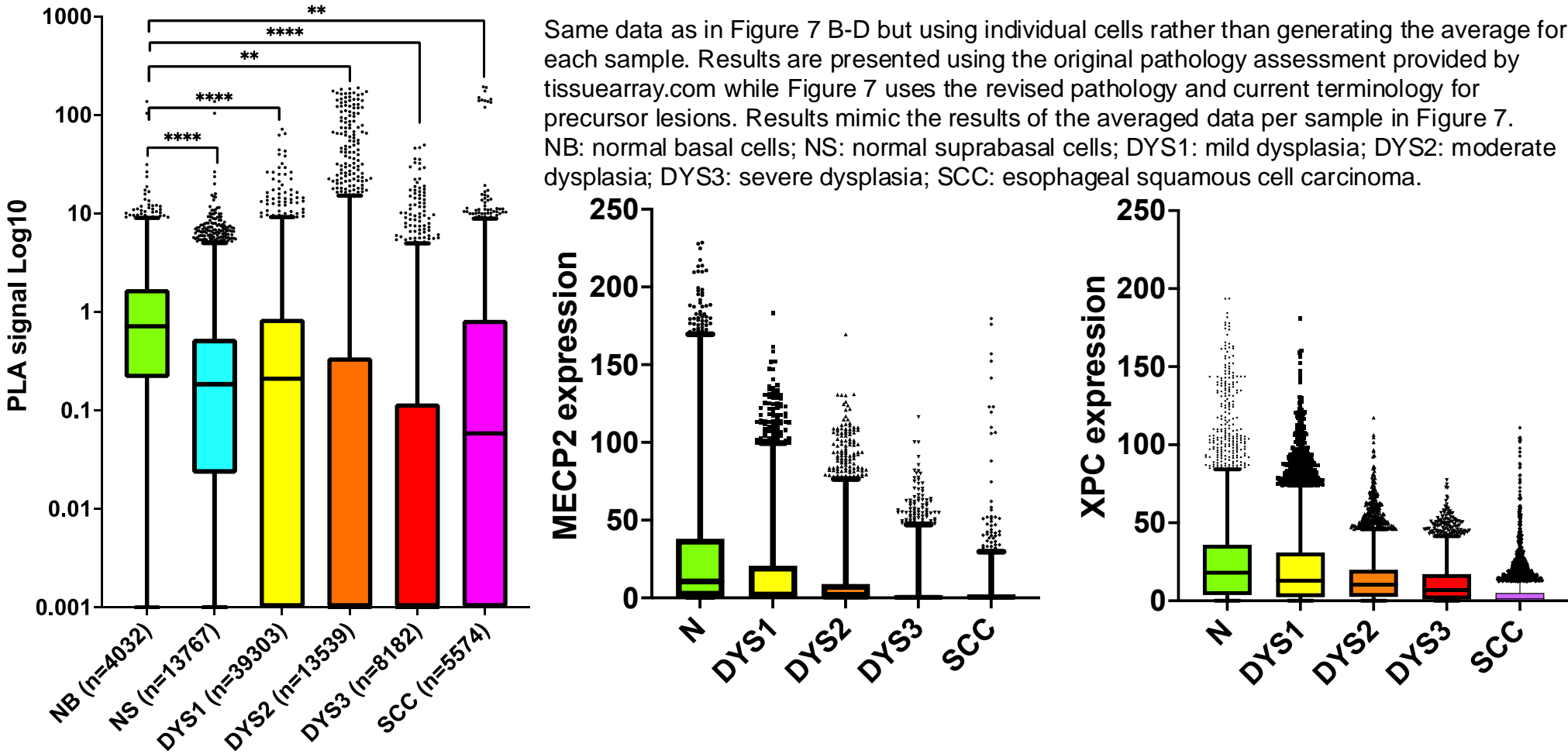

Supplement: Supplementary file 1 [file ijms-26-03144-s001.zip › Supplementary Figure S4 PLA.pdf]
